# Ulososides and Urabosides — Triterpenoid Saponins from the Caribbean Marine Sponge *Ectyoplasia ferox*

**DOI:** 10.3390/molecules18032598

**Published:** 2013-02-27

**Authors:** Jhonny Colorado, Diana Muñoz, Diana Marquez, Maria Elena Marquez, Juan Lopez, Olivier P. Thomas, Alejandro Martinez

**Affiliations:** 1 Grupo de Investigación Productos Naturales Marinos, Facultad de Química Farmacéutica, Universidad de Antioquia, A.A 1226, Medellín, Colombia; E-Mails: dmarquez@farmacia.udea.edu.co (D.M.); amart@farmacia.udea.edu.co (A.M.); 2 Unidad de Investigación e Innovación, Humax Pharmaceutical S.A. Itagüí, Colombia; 3 Grupo de Biotecnología Animal, Universidad Nacional de Colombia, Medellín, Colombia; E-Mails: ingbiodianacaro@gmail.com (D.M.); memarque@unal.edu.co (M.E.M.); jblopez@unal.edu.co (J.L.); 4 Nice Institute of Chemistry – PCRE, UMR 7272 CNRS, University of Nice Sophia-Antipolis, Faculté des Sciences, Parc Valrose 06108 Nice, France

**Keywords:** *Ectyoplasia ferox*, marine sponge, saponins, urabosides, ulososides

## Abstract

Three new triterpene glycosides, named ulososide F (**1**), urabosides A (**2**) and B (**3**), together with the previously reported ulososide A (**4**), were isolated from the Caribbean marine sponge *Ectyoplasia ferox*. Their structures were elucidated using extensive interpretation of 1D and 2D-NMR data, as well as HRESIMS. The aglycon of all compounds is a rare 30-norlonastane and the sugar residues were identified after acid hydrolysis and GC analyses. Cytotoxicities of the isolated compounds were evaluated against Jurkat and CHO cell lines by a MTT *in vitro* assay as well as a hemolysis assay. Unexpectedly, all these saponin derivatives showed very low activity in our bioassays.

## 1. Introduction

Secondary metabolites play critical roles in the chemical defense systems of many marine organisms and they are frequently involved in the organization of benthic communities. Marine sponges are already well known as producers of such secondary metabolites with ecological roles, especially in the Caribbean region, where they can dominate some ecosystems [1]. The high structural diversity of these secondary metabolites has been associated to the lack of physical defenses forcing them to develop chemical defenses to be used, for example, to deter predation [2]. The original chemical diversity of these metabolites have also found applications in the therapeutic field and this research is still in its infancy with the first marketing of the sponge-inspired eribulin mesylate as an anticancer agent by the Eisai company in 2011 [3].

We decided to embark on a program aiming at describing the pharmaceutical potential of secondary metabolites produced by dominant sponges of the South-Eastern Caribbean Sea, a region with a difficult access and thus rarely studied in this context. *Ectyoplasia ferox* (Duchassaing & Michelotti, 1864) (Poecilosclerida, Raspailiidae) was selected for a full chemical study due to its high abundance in the Urabá Gulf, our region of interest. This species has also been described at sites in the Northern part of the Caribbean, like Florida or even the Bahamas, and our chemical study will therefore also shed light on some possible differences in the secondary metabolome induced by geographical and/or environmental factors. The place of *E. ferox* in the order Poecilosclerida has been demonstrated, but the absence of guanidinic alkaloids was intriguing, as they are usually recognized as chemotaxonomic markers of this group [4,5,6]. This common sponge has also been proven as a good model for studies concerning the transfer of the microbial communities during the reproduction [7,8]. From the four species described so far within this genus, chemical studies have only been undertaken on *E. ferox* and this point also raised our interest. Triterpenoid saponins, named ectyoplasides and feroxosides, were identified as major chemical components of this species [9,10], but polar lipids were also reported [11,12,13]. These compounds were suggested to possess ecological roles, but also to have pharmaceutical potential.

**Figure 1:**
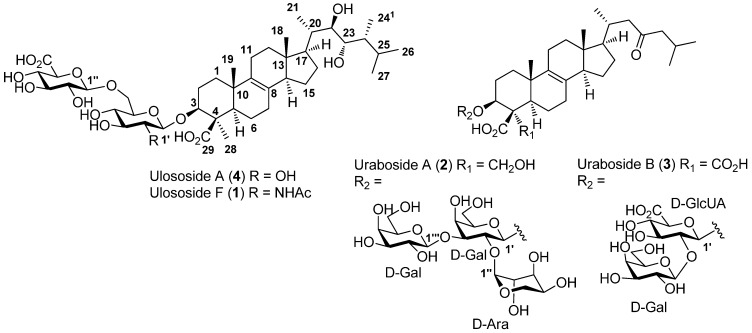
Structures of saponins **1**–**4** from *E. ferox*.

Triterpenoid and steroidal glycosides are mainly found in the marine environment in echinoderms where they can act as chemical defense. However, few chemical studies have also underlined their presence in marine sponges, mostly from the genera *Erylus*, *Mycale*, *Melophlus* [14,15] and more recently from a *Pandaros* species (Poecililosclerida, Microcionidae) [16,17,18]. We report herein the results of our chemical study on a specimen of *Ectyoplasia ferox* collected in the Urabá Gulf (Colombia) and the isolation and structure elucidation of four triterpenoid saponins **1**–**4** which clearly differ from the previously isolated ectyoplasides and feroxosides (Figure 1).

## 2. Results and Discussion

### 2.1. Isolation and Structure Elucidation

The sponge specimen was lyophilized, ground, and repeatedly extracted with a 1:1 CH_2_Cl_2_/MeOH mixture. The combined extracts were filtered, concentrated under vacuum and fractionated by reversed phase column liquid chromatography with solvents of decreasing polarities (H_2_O, MeOH and CH_2_Cl_2_). The methanol fraction was further subjected to repeated HPLC purifications yielding the new ulososide F (**1**) and urabosides A (**2**) and B (**3**), together with the known compound uloloside A (**4**) (Figure 1). These compounds were identified by combined spectroscopic methods and comparisons of NMR data with literature [10,19,20,21].

Compound **1** was isolated as a white amorphous solid, with a molecular formula assigned as C_44_H_71_NO_16_ on the basis of combined HRESIMS (*m/z* 892.46222 [M+Na]^+^) and ^13^C-NMR data. The saponin nature of this compound was evidenced in the ^1^H-NMR spectrum of **1** by the presence of characteristic terpenoid methyl singlets (*δ*_H_ between 0.6 and 1.5) together with sugar residues (*δ*_H_ between 3.1 and 5.0). The portion of the ^1^H-NMR spectrum corresponding to the aglycon evidenced seven methyl signals that were reminiscent of a 29-functionalized 24-methyl-30-norlanostane at *δ*_H_ 0.67 (s, H_3_-18), 0.85 (d, H_3_-24^1^), 0.93 (d, H_3_-21), 0.95 (d, H_3_-26), 0.96 (d, H_3_-27), 1.01 (s, H_3_-19), 1.34 (s, H_3_-28) (Table 1) [22]. The presence of a fused double bond at C-8/C-9 was mainly inferred from the key H_3_-19/C-9 HMBC correlation. Two alcohols and a methyl were placed at C-22, C-23 and C-24 of the side-chain respectively after inspection of the COSY spectrum and characteristic signals at *δ*_H_ 3.51 (m, H-22), 3.68 (dd, H-23), 1.56 (m, H-24) and *δ*_C_ 73.4 (CH, C-22), 72.2 (CH, C-23), 41.0 (CH, C-24). The structure was then very similar to the previously isolated ulososides isolated from an *Ulosa* sp. sponge collected in Madagascar [19,20,21]. In fact, **1** shares exactly the same aglycon as ulososides A, C, D and E due to very close chemical shifts, even if all NMR spectra were reported in C_5_D_5_N in the literature. We just changed the numbering replacing the C-28 methyl described in the literature by a C-24^1^ methyl at C-24, thus respecting the IUPAC recommendations for this family of triterpenoids. The relative configuration of the tetracycle was confirmed by NOESY correlations and ^3^*J* scalar coupling interpretation [23]. We decided to assess the relative configuration of the asymmetric centers at C-22, C-23 and C-24 of the side-chain using a close inspection of the NMR data reported for the closely related and well known brassinosteroids [23,24,25]. 

**Table 1:** ^1^H (500 MHz) and ^13^C-NMR (125 MHz) data for the aglycon of **1**, **2**, and **3** (in CD_3_OD).

Position	1		2		3	
	*δ*_H_, mult. (*J* in Hz)	*δ*_C_, mult.	*δ*_H_, mult. (*J* in Hz)	*δ*_C_, mult.	*δ*_H_, mult. (*J* in Hz)	*δ*_C_, mult.
1a	1.86, m	37.4, CH_2_	1.83, m	38.2, CH_2_	1.87	37.8, CH_2_
1b	1.32, m		1.27, m		1.35	
2a	2.38, qd (13.5, 2.5)	28.2, CH_2_	2.57, m	28.1, CH_2_	2.23, d (12.0)	28.0, CH_2_
2b	2.05, m		2.01, m		2.06, m	
3	3.28 ª	89.8, CH	3.80, m	81.3, CH	4.00, dd (12.0, 4.0)	87.5, CH
4		50.5, C		55.9, C		63.8, C
5	1.33, m	53.2, CH	1.78, m	44.6, CH	1.98, m	50.6, CH
6a	1.94, m	21.2, CH_2_	1.92, m	20.8, CH_2_	2.13, m	23.8, CH_2_
6b	1.66, m		1.54, m		1.54, m	
7a	2.07, m	29.8, CH_2_	2.08, m	28.8, CH_2_	2.01, m	29.8, CH_2_
7b	1.93, m		2.01, m		1.92, m	
8		129.1, C		128.9, C		129.8, C
9		137.3, C		137.5, C		136.3, C
10		38.5, C		38.7, C		37.7, C
11a	2.17, m	23.3, CH_2_	2.15, m	23.2, CH_2_	2.14, m	23.1, CH_2_
11b	2.08, m		2.08, m		2.10, m	
12a	2.00, m	38.4, CH_2_	1.98, m	38.4, CH_2_	1.98, m	38.3, CH_2_
12b	1.43, m		1.43, m		1.43, m	
13		43.4, C		43.4, C		43.5, C
14	2.11, m	53.3, CH	2.09, m	53.2, CH	2.10, m	53.2, CH
15a	1.63, m	24.8, CH_2_	1.63, m	24.7, CH_2_	1.63, m	24.8, CH_2_
15b	1.37, m		1.34, m		1.34, m	
16a	1.92, m	29.1, CH_2_	1.91, m	29.1, CH_2_	1.90, m	29.1, CH_2_
16b	1.36, m		1.36, m		1.33, m	
17	1.57, m	52.5, CH	1.22, m	56.1, CH	1.22, m	56.1, CH
18	0.67, s	11.8, CH_3_	0.67, s	11.7, CH_3_	0.68, s	11.8, CH_3_
19	1.01, s	18.9, CH_3_	1.02, s	18.9, CH_3_	0.98, s	18.9, CH_3_
20	1.89, m	37.9, CH	1.99, m	34.2, CH	1.99, m	34.2, CH
21	0.93, d (6.8)	12.4, CH_3_	0.93, d (6.8)	20.3, CH_3_	0.93, d (6.8)	20.3, CH_3_
22a	3.51, br d (9.5)	73.4, CH	2.49, dd (16.0, 3.5)	51.0, CH_2_	2.49, dd (16.0, 3.5)	51.0, CH_2_
22b			2.17, m		2.17, m	
23	3.68, dd (9.5, 1.5)	72.2, CH		213.8, C		213.8, C
24	1.56, m	41.0, CH	2.30, d (6.8)	53.6, CH_2_	2.30, d (6.8)	53.6, CH_2_
24^1^	0.85, d (6.9)	10.0, CH_3_				
25	1.62, m	32.2, CH	2.08, m	25.8, CH	2.08, m	25.8, CH
26	0.95, d (6.6)	21.6, CH_3_	0.90, d (6.6)	22.8, CH_3_	0.90, d (6.6)	22.8, CH_3_
27	0.96, d (6.6)	21.2, CH_3_	0.92, d (6.6)	22.8, CH_3_	0.92, d (6.6)	22.8, CH_3_
28a	1.35, s	24.8, CH_3_	3.96, d (11.0)	60.7, CH_2_		^b^
28b			3.89, d (11.0)			
29		177.8, C		176.9, C		175.2, C

^a^ Overlapped with the CD_3_OD residual peak; ^b^ Not seen.

**Figure 2:**
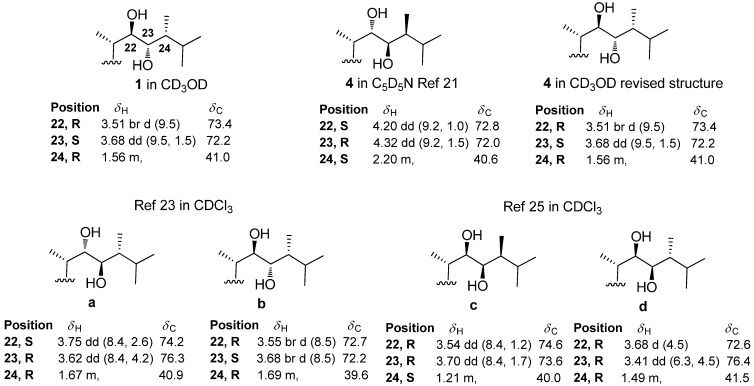
Comparison of NMR data of the aglycon side-chain.

It appears that ^1^H and ^13^C-NMR data are fully consistent with a 22*R*, 23*S* and 24*R* configurations assuming a usual absolute configuration at C-20 (Figure 2). Additionally, we then propose to change the configurations for ulososides A, C, D and E that were tentatively assigned as 22*S*, 23*R* and 24*S*.

NMR spectra indicated the disaccharide nature of compound **1**. For the glycosidic part of the molecule, two sugar residues were evidenced by the signals corresponding to two anomeric carbons at *δ_C_* 104.8 (CH, C-1′), and 105.1 (CH, C-1′′) and protons at *δ*_H_ 4.56 (d, *J* = 8.1 Hz, H-1′) and 4.49 (d, *J* = 7.8 Hz, H-1′′), with coupling constants at *J* = 8.1 and 7.8 Hz suggesting a *β* anomeric configuration (Table 2). The first sugar residue was identified as a *N*-acetylglucosamine on the basis of the ^13^C chemical shifts and coupling constant values, as well as a comparison with ulososide D-NMR data [19]. A H-1′/C-3 HMBC correlation placed this sugar at C-3. Compared to ulososide D an additional glucuronic acid was branched at C-6′ on the basis of a H-1′′/C-6′ HMBC correlation. The glucuronic acid was ascertained by interpretation of coupling constant values and the presence of a H-5′′/C-6′′ HMBC correlation. The D absolute configurations of both sugar residues were determined by GC after derivatization and comparison with standard sugars [26].

Compound **2** was isolated as a white, amorphous solid with a molecular formula of C_46_H_74_O_19_ on the basis of the pseudomolecular ion at *m/z* 953.47165 [M + Na]^+^ by HRESIMS analysis. The aglycon of **2** differed from **1** in two main sites. First, changes in the ^1^H and ^13^C-NMR chemical shifts of the signals corresponding to C-3, C-4 and C-5 indicated a change in the substitution pattern at C-4. In the ^1^H-NMR spectrum, the methyl at C-28 for **1** was replaced by an AB system in **2** at *δ*_H_ 3.96 (d, *J* = 11.0 Hz, H-28a) and 3.89 (d, *J* = 11.0 Hz, H-28b) which was reminiscent of a methylene oxy at C-4 (Table 1). This assumption was confirmed by comparison with the corresponding signals found for ulososide B [20]. Comparison of the chemical shifts between both compounds and a clear H-29/H-3 NOESY correlation allowed us to assign the relative configuration at C-3 and C-4. The second modification was observed for the signals corresponding to the side-chain protons and carbons. Indeed, the signals corresponding to the vicinal diol of **1** were clearly absent as well as the one corresponding to the methyl at C-24. In contrast, a ketone was unambiguously placed at C-23 due to the ^13^C-NMR signal at *δ*_C_ 213.8 (C, C-23) and the key H-22a, H-22b, H-24/C-23 HMBC correlations. Interestingly, this side-chain was identical to the one of some pandarosides, steroidal saponins found in another Poecilosclerida sponge of the genus *Pandaros* [18,27].

**Table 2:** ^1^H (500 MHz) and ^13^C-NMR (125 MHz) data for the sugars of **1**, **2**, and **3** (in CD_3_OD).

Position	1		2		3	
	*δ*_H_, mult. (*J* in Hz)	*δ*_C_, mult.	*δ*_H_, mult. (*J* in Hz)	*δ*_C_, mult.	*δ*_H_, mult. (*J* in Hz)	*δ*_C_, mult.
1′	4.56, d (8.1)	104.8, CH	4.56, d (7.9)	105.3, CH	4.51, d (7.6)	104.6, CH
2′	3.61, dd (8.1, 9.5)	58.0, CH	3.90, m	74.3, CH	3.70, t (7.8)	79.8, CH
3′	3.53, t (9.5)	75.5, CH	3.79, m	86.7, CH	3.55, m	77.5, CH
4′	3.28 ª	72.1, CH	4.15, br d (3.0)	70.3, CH	3.56, m	73.0, CH
5′	3.49, ddd (9.4, 6.3, 1.6)	77.0, CH	3.53, m	76.1, CH	3.80, d	76.6, CH
6′a	4.11, dd (11.8, 1.8)	70.2, CH_2_	3.73, m	62.6, CH_2_		172.2, C
6′b	3.79, dd (11.8, 6.4)					
-CO-CH_3_	1.93, s	23.0, CH_3_				
-CO-CH_3_		173.5, C				
1′′	4.49, d (7.8)	105.1, CH	5.59, d (3.2)	100.2, CH	4.57, d (8.1)	104.6, CH
2′′	3.26, dd (9.3, 7.8)	74.8, CH	3.78, m	70.4, CH	3.60, dd (9.3, 8.1)	73.7, CH
3′′	3.40, t (9.3)	77.5, CH	3.82, m	70.8, CH	3.44, dd (9.3, 3.0)	75.1, CH
4′′	3.53, m	73.2, CH	3.76, m	70.3, CH	3.76, d (3.0)	71.2, CH
5′′a	3.78, d (9.6)	76.6, CH	4.07, d (12.8)	64.9, CH_2_	3.53, m	76.9, CH
5′′b			3.42 (m)			
6′′a		172.5, C			3.88, dd, (11.7, 7.6)	63.0, CH_2_
6′′b					3.65, dd, (11.7, 3.5)	
1′′′			4.50, d (7.8)	106.1, CH		
2′′′			3.61, m	72.9, CH		
3′′′			3.46, m	75.1, CH		
4′′′			3.82, m	70.4, CH		
5′′′			3.53, m	76.8, CH		
6′′′			3.70, m	62.6, CH		

^a^ Overlapped with the CD_3_OD residual peak.

The osidic part of this saponin was shown to contain three sugar residues as evidenced by the signals of three anomeric proton doublets at *δ*_H_ 4.56 (d, H-1′), 5.59 (d, H-1′′) and 4.50 (d, H-1′′′) and the corresponding anomeric carbons at *δ*_C_ 105.3 (CH, C-1′), 100.2 (CH, C-1′′) and 106.1 (CH, C-1′′′) observed in the HSQC spectrum. The three sugar residues were confidently located at C-3, C-2′ and C-3′ on the basis of H-1′/C-3, H-1′′/C-2′ and H-1′′′/C-3′ HMBC correlations. Two hexoses were deduced from the signals at *δ*_H_ 3.73 (m, H-6′), 3.70 (m, H-6′′′) and *δ*_C_ 62.6 (CH_2_, C-6′ and C-6′′′). The third sugar residue was found to be a pentose due to the presence of the signals at *δ*_H_ 4.07 (d, H-5′′a), 3.42 (m, H-5′′b) and *δ*_C_ 64.9 (CH_2_, C-5′′).

The ring sizes and identity of these sugar residues were determined by 2D-NMR experiments and by comparison of chemical shifts of protons and carbons of each monosaccharide with literature citations [28,29]. After the assignment of the individual NMR signals for these sugar units by extensive COSY, TOCSY, and HSQC analyses, HMBC experiments showed long-range correlations at H-1′/C-5′, H-5′/C-1′, H-5b′′/C-1′′, H-1′′′/C-5′′′, and H-5′′′/C-1′′′, thus revealing the pyranose nature of all three residues. The large coupling constant values for the doublets assigned to H-1′ and H-1′′′ (*J* = 7.9 and 7.8 Hz) implied that these hexoses were connected through *β*-glycosidic linkages, while the smaller coupling constant value for H-1′′ (*J* = 3.2 Hz) suggests also a *β* linkage for this arabinose residue. The absolute configurations of these three residues was ascertained after acidic hydrolysis, derivatization into chiral butylated derivatives and GC analysis which indicated a D-galactose at C-3, a D-arabinose at C-2′ and a second D-galactose at C-3′.

Compound **3** was obtained as a white, amorphous powder. The positive ion at *m*/*z* 849.38367 [M+Na]^+^ in HRESIMS and ^13^C-NMR data indicated an empirical molecular formula of C_41_H_62_O_17_. The signals corresponding in the NMR spectra of **3** were closely related with the signals of **2** indicating a close relationship between these two compounds. The sole difference appears for the signals corresponding to the positions C-3, C-4 and C-5 (Table 1). The absence of the AB system assigned to the primary alcohol of 1 at C-4 suggested a diverse functionalization at this position. Even if the corresponding signal was not observed in the ^13^C neither in the HMBC NMR spectra, the molecular formula was only consistent with a presence of a second carboxylic acid at this position. This is the first example of such a substitution pattern at C-4 for a terpenoid and then no comparison with literature data was available. Nevertheless, NMR modeling with ChemDraw was fully consistent with the observed chemical shifts in ^1^H-NMR and more importantly in ^13^C-NMR, especially for *δ*_C_ 63.8 (C, C-4), thus confirming this assumption.

Two hexose residues were observed in **3** which were identified as a first glucuronic acid linked at C-3 to the aglycon due to the presence of a H-1′/C-3 HMBC correlation. Coupling constant values and a clear H-5′/C-6′ HMBC correlation confirmed the *β*-glucuronic acid. The second sugar residue was linked at C-2′ to this first residue on the basis of a H-1′′/C-2′ HMBC correlation. This second residue was found to be a galactose unit due to the coupling constant values of the signal at *δ*_H_ 3.76 (d, *J* = 3.0 Hz, H-4′′). Furthermore, the value of *J* = 8.1 Hz for the coupling constant of H-1′′ ascertained a *β* linkage with the first residue. Using the same process used for **2** we confirmed the relative configurations and proposed a D absolute configuration for both residues.

### 2.2. Bioactivity

No significant cytotoxicity against two cell lines (Jurkat and CHO cells) or hemolytic activity was detected below 50 µM for any of the isolated compounds. Only compound **3** exhibited a low cytotoxicity, with an IC_50_ value of 100 µM. Other compounds were considered inactive, with IC_50_ > 100 µM, and with cytotoxicities below 30% up to 100 µM. Interestingly, compounds **1** to **4** did not induce hemolysis, which is the major known adverse effect of this kind of molecules and the major obstacle for clinical trials progress. Previous studies on the bioactivities of triterpene glycosides from *E. ferox* reported moderate cytotoxicity against J774 (murine monocyte-macrophage), WEHI164 (murine fibrosarcoma), and P388 (murine leukemia) cell lines at IC_50_ ranging from 8.5 to 19 μg/mL [9,10]. The biological activities of our new derivatives are significantly lower than those exhibited by the previously isolated ectyoplasides and feroxosides and consequently it suggests a key role of the aglycone in the cytotoxicity of these compounds. The assessment of detergent-like properties by the hemolysis assay demonstrated that there is no significant effect of our compounds.

## 3. Experimental

### 3.1. General Procedures

Optical rotations were measured on Perkin Elmer 343 polarimeter equipped with a 10 cm microcell. IR spectra were obtained with a Perkin–Elmer Paragon 1000 FT–IR spectrometer. High resolution mass spectra (HRESIMS) were obtained from a LTQ Orbitrap mass spectrometer (Thermo Finnigan). NMR experiments were performed on a Bruker Advance 500 MHz spectrometer. Chemical shifts (*δ* in ppm) are referenced to the carbon (*δ*_C_ 49.0) and residual proton (*δ*_H_ 3.31) signals of CD_3_OD, the solvent with multiplicity (s singlet, d doublet, t triplet, m multiplet). Column chromatography was performed using RP18 stationary phase (40–63 μm, Merck). HPLC separation and purification were carried out on an Agilent1100 system equipped with an Agilent UV detector and coupled with a Varian 385-ELSD. For GC-MS analysis of the derivatized sugar residues an Agilent 6890N GC interfaced to a 5975B MSD was used. TLC was performed with Kieselgel 60 F_254_ (Merck glass support plates) and spots were detected after spraying with 10% H_2_SO_4_ in EtOH reagent and heating. The OD_570_ nm absorbance for cytotoxicity evaluation was measured with a Thermo Scientific Multiskan® Spectrum instrument.

### 3.2. Biological Material

The marine sponge was collected off Caribbean Sea, Colombia, in October 2008 by SCUBA diving (Urabá Gulf 8°40′14′′N, 77°21′28′′W). A voucher specimen (INV-POR 0335) identified by Sven Zea, has been deposited in the sponge collection of Museo de Historia Natural Marina de Colombia, Invemar. The sponge was kept frozen from collection until the extraction process.

### 3.3. Extraction and Isolation

The frozen sponge (350 g wet) was cut into pieces of about 1 cm^3^ and extracted with 1:1 MeOH/CH_2_Cl_2_ (600 mL, 24 h) at room temperature yielding 3.6 g of crude extract after solvent evaporation. The crude extract was fractionated by RP-C_18_ column chromatography (elution with a decreasing polarity gradient of H_2_O/MeOH from 70:30 to 0:100, then MeOH/CH_2_Cl_2_ from 100:0 to 0:100). The third fraction (175.5 mg) from 10 collected (MeOH 100%) was then subjected to RP semi-preparative HPLC (Phenomenex, Gemini C_6_-phenylhexyl 110 Å, 250 × 10 mm, 5 μm) with a gradient of H_2_O/acetonitrile/TFA (flow 3.0 mL·min^−1^ from 60:40:0.1 to 45:55:0.1) to yield pure compound **2** (tr: 23.1 min; 1.4 mg); whereas the second fraction (122.5 mg) was purified with an isocratic mobile phase of H_2_O/Acetonitrile/TFA (flow 3.0 mL·min^−1^, 55:45:0.1) to afford pure metabolites **1**, **4** and **3** (tr: 15.2, 16.8 and 24.1 min; 4.5, 3.6 and 2.9 mg, respectively).

### 3.4. Isolated Compounds

*(22R,23S,24R)-3β-O-(β-D-Glucopyranosyluronic acid-(1→6)-2-acetamido-2-deoxy-β-D-gluco-pyranosido)-3,22,23-trihydroxy-24-methyl-30-norlanost-8(9)-en-29-oic acid* (*Ulososide F*, **1**). White amorphous solid; [α]^20^_D_ −4.0 (c 0.036, MeOH); IR (thin film): γ_max_ 3392, 2956, 2874, 1721, 1669 cm^−1^; ^1^H-NMR and ^13^C-NMR see Table 1, Table 2; HRESIMS (+): *m/z* 892.46222 [M+Na]^+^ (calcd for C_44_H_71_NNaO_16_, 892.46651, Δ −4.8 ppm).

*3β-O-[β-D-Arabinopyranosyl-(1→2)-(β-D-galactopyranosyl-(1→3))-β-D-galactopyranosid]-3,28-dihydroxy-23-oxolanost-8(9)-en-29-oic acid* (*Uraboside A*, **2**). White amorphous solid; [α]^20^_D _−330 (c 0.013, MeOH); IR (thin film): γ_max_3392, 2988,1625, 1072 cm^−1^; ^1^H-NMR and ^13^C-NMR see Table 1, Table 2; HRESIMS (+): *m/z* 953.47327 [M+Na]^+^ (calcd for C_46_H_74_NaO_19_, 953.47165, Δ −1.7 ppm).

*3β-O-[β-D-Galactopyranosyl-(1→2)-β-D-glucopyranosiduronic acid]-3-hydroxy-23-oxolanosta-8(9)-ene-28,29-dioic acid* (*Uraboside B*, **3**). White amorphous solid; [α]^20^_D_−130 (c 0.027, MeOH); IR (thin film): γ_max _3409, 2920, 1712, 1674 cm^−1^; ^1^H-NMR and ^13^C-NMR see Table 1, Table 2; HRESIMS (+): *m/z* 849.38367 [M+Na]^+^ (calcd for C_41_H_62_O_17_Na, 849.38792, Δ −5.0 ppm).

*(22R,23S,24R)-3β-O-(β-D-Glucuronopyranosyl-(1→6)-β-D-glucopyranosyl)-3,22,23-trihydroxy-24-methyl-30-norlanost-8(9)-en-29-oic acid* (*Ulososide A*, **4**). White amorphous solid; [α]^20^_D_ −9.5 (c 0.23, MeOH); IR (thin film): γ_max_ 3425, 1715, 1648 cm^−1^; ^1^H-NMR (500 MHz, CD_3_OD) 1.84 (m), 1.36 (m), 2.41 (m), 2.03 (m), 3.30 (m), 1.34 (m), 1.97 (m), 1.71 (m), 2.07 (m), 1.93 (m), 2.17 (m), 2.08 (m), 1.99 (m), 1.46 (m), 2.11 (m), 1.64 (m), 1.34 (m), 1.99 (m), 1.44 (m), 1.57 (m), 0.67 (s), 1.01 (s), 1.90 (m), 0.93 (d, 6.9 Hz), 3.51 (m), 3.66 (dd, 1.6 Hz, 9.7 Hz), 1.55 (m), 1.62 (m), 0.97 (d, 6.6 Hz), 0.95 (d, 6.6 Hz), 0.85 (d, 6.9 Hz), 1.44 (s), 4.32 (d, 7.8 Hz), 3.25 (m), 3.47 (m), 3.27 (m), 3.53 (m), 4.08 (s, b), 3.77 (m), 4.47 (d, 7.8 Hz), 3.25 (m), 3.39 (m), 3.51 (m), 3.81 (d, 9.6 Hz); ^13^C-NMR (125 MHz, CD_3_OD) 37.7 (C-1), 28.5 (C-2), 89.4 (C-3), 50.7 (C-4), 53.6 (C-5), 21.3 (C-6), 29.9 (C-7), 129.3 (C-8), 137.1 (C-9), 23.2 (C-11), 38.5 (C-12), 43.3 (C-13), 53.3 (C-14), 24.8 (C-15), 29.1 (C-16), 52.5 (C-17), 11.9 (C-18), 18.6 (C-19), 38.0 (C-20), 12.3 (C-21), 73.2 (C-22), 72.2 (C-23), 41.0 (C-24), 32.2 (C-25), 21.6 (C-26), 21.2 (C-27), 10.0 (C-28), 179.0 (C-29), 24.3 (C-30), 107.2 (C-1′), 75.3 (C-2′), 77.0 (C-3′), 71.7 (C-4′), 73.2 (C-5′), 70.2 (C-6′), 105.1 (C-1′′), 74.9 (C-2′′), 77.9 (C-3′′), 73.5 (C-4′′), 76.8 (C-5′′), 171.2 (C=O); HRESIMS (+): *m/z* 851.43524 [M + Na]^+^ (calcd for C_42_H_68_NaO_16_, 851.43996, Δ −5.6 ppm).

### 3.5. Acidic Hydrolysis and GC-MS Analysis of the Butylated Sugar Derivatives

D- and L- determination was performed using combined gas chromatography/mass spectrometry (GC/MS) of the butylated derivatives of the monosaccharides produced from the compounds **1**–**4** by acidic hydrolysis. The sample materials were placed into individual test tubes. To this, aqueous 2 M TFA (400 µL) was added. The tubes were then placed at 121 °C for 1.5 h. Once cooled, the sample was dried under nitrogen. The dried samples were then re-*N*-acetylated with pyridine and acetic anhydride in methanol. After drying, the samples were butylated using *S*-(+)-2-butanol or *R*-(+)-2-butanol (Sigma) for 16 h at 80 °C. The samples were per-*O*-trimethylsilylated by treatment with Tri-Sil (Pierce) at 80 °C (0.5 h). GC/MS analysis of the butylated derivatives was performed using a Supelco EC-1 fused silica capillary column (30m × 0.25 mm ID). Individual standards of all the detected residues were analyzed in parallel with each sample. Comparison of retention times allowed for the elucidation of the specific conformation of each monosaccharide in the compounds. All residues were of D configuration.

### 3.6. Cell Cultures

Jurkat and CHO-K1 cells were cultured at 37 °C in a humidified 5% CO_2_ atmosphere in 24 cm^2^ cell culture flasks in RPMI 1640 medium supplemented with 5% heat inactivated fetal bovine serum, 2 mM L-glutamine, free of antibiotics.

### 3.7. MTT Test

The purity of all tested compounds was found to be higher than 96% based on HPLC-ELSD analysis. Compounds at final concentrations of 1 and 10 μM was added to 100 μL of the CHO and Jurkat cells suspension (1.0 × 10^4^/well for Jurkat cells and 8.0 × 10^3^/well for CHO cells in RPMI 1640 medium with 5% of FBS) onto wells. Cells were treated with each sample for 24 h at 37 °C in 5% CO_2_. After treatment, the viability of cells was evaluated by the MTT assay. MTT reagent was added to each plate, and after 4 h of incubation, 100 µL of acidic isopropyl alcohol mixture (50 mL Triton X-100, 4 mL of 30% HCl and 446 mL of isopropyl alcohol) was added to dissolve the water-insoluble formazan salt for overnight. The OD_570_ nm absorbance was measured. Each concentration was tested by triplicated and unexposed cells were regarded as 100% viable [30].

### 3.8. Hemolysis Assay

The method was adapted from van Duick *et al.* [31] and from Taniyama *et al.* [32]. Blood was obtained from healthy young male donors. The red blood cells (RBCs) were washed three times and resuspended in sterile RPMI 1640 medium (S&A) PBS to give about 15 *×* 10^6^ cells per mL and further processed [31]. The erythrocytes were incubated with compounds in a range 100–200 μM for 1 h at 37 °C. After centrifugation of the nonhemolysed erythrocytes (800 rpm, 5 min), the absorbance of the released hemoglobin in the wavelength 540 nm was measured. The percentage of hemolysis was determined by comparing the absorbance of hemoglobin at 540 nm released from the RBCs in the presence of each compound. The positive control (100% hemolysis) was determined by the amount of hemoglobin released from 15 *×* 10^6^ RBCs after 1h of incubation with 0.1% of Tween-20.

## 4. Conclusions

The structures of glycoconjugates found in some marine invertebrates are used as chemotaxonomic markers at different taxonomic levels (species, genus, and subfamily) [14]. As established earlier, glycoside terpenoids belonging to the same taxon show characteristic structural features that were found to be independent of the collection site and season. Glycosides from *E. ferox* collected in different geographic areas demonstrate structural similarity to each other. They contain related carboxylated aglycons of the lanostane type and their carbohydrate chains often include galactose and glucuronic acid units. It is interesting to note that the structures of the triterpenoid saponins isolated in this study differed from those isolated from the same species, but collected earlier in the Northern Caribbean. The most distinguishing feature is undoubtedly the presence of carboxy groups at C-4 of the aglycon. Difficulties in the isolation and structure elucidation of these compounds may also explain our finding in a previously studied species. Saponins are then confirmed as common compounds in marine sponges and especially in sponges belonging to the order Poecilosclerida. This can be of taxonomical significance and should trigger further chemical studies in this group.

## Supplementary Materials

Supplementary materials can be accessed at: http://www.mdpi.com/1420-3049/18/3/2598/s1.
