# Supplementary material for: Ulososides and Urabosides — Triterpenoid Saponins from the Caribbean Marine Sponge Ectyoplasia ferox
Source: Molecules. 2013 Feb 27;18(3):2598–610. doi: 10.3390/molecules18032598 (PMC6270507; doi:10.3390/molecules18032598)

Supporting Information

**Page**	**Contents**
S1	^{1}H-NMR spectrum of **1** (500 MHz, CD_3OD)
S2	^{13}C-NMR spectrum of **1** (125 MHz, CD_3OD)
S3	COSY spectrum of **1** (500 MHz, CD_3OD)
S4	HSQC spectrum of **1**
S5	HMBC spectrum of **1**
S6	TOCSY spectrum of **1**
S7	HRESIMS of **1**
S8	^{1}H-NMR spectrum of **2** (500 MHz, CD_3OD)
S9	^{13}C-NMR spectrum of **2** (125 MHz, CD_3OD)
S10	COSY spectrum of **2** (500 MHz, CD_3OD)
S11	HSQC spectrum of **2**
S12	HMBC spectrum of **2**
S13	Zoom of HMBC spectrum of **2**
S14	NOESY spectrum of **2**
S15	HRESIMS spectrum of **2**
S16	^{1}H-NMR spectrum of **3** (500 MHz, CD_3OD)
S17	^{13}C-NMR spectrum of **3** (125 MHz, CD_3OD)
S18	COSY spectrum of **3** (500 MHz, CD_3OD)
S19	HSQC spectrum of **3**
S20	HMBC spectrum of **3**
S21	Zoom of the HMBC spectrum of **3**
S22	HRESIMS of **3**
S23	^{1}H-NMR spectrum of **4** (500 MHz, CD_3OD)
S24	^{13}C-NMR spectrum of **4** (125 MHz, CD_3OD)
S25	COSY spectrum of **4** (500 MHz, CD_3OD)
S26	HSQC spectrum of **4**
S27	HMBC spectrum of **4**
S28	TOCSY spectrum of **4**
S29	HRESIMS of **4**

Figure S1. ^{1}H-NMR spectrum of **1** (500 MHz) in CD_3OD.

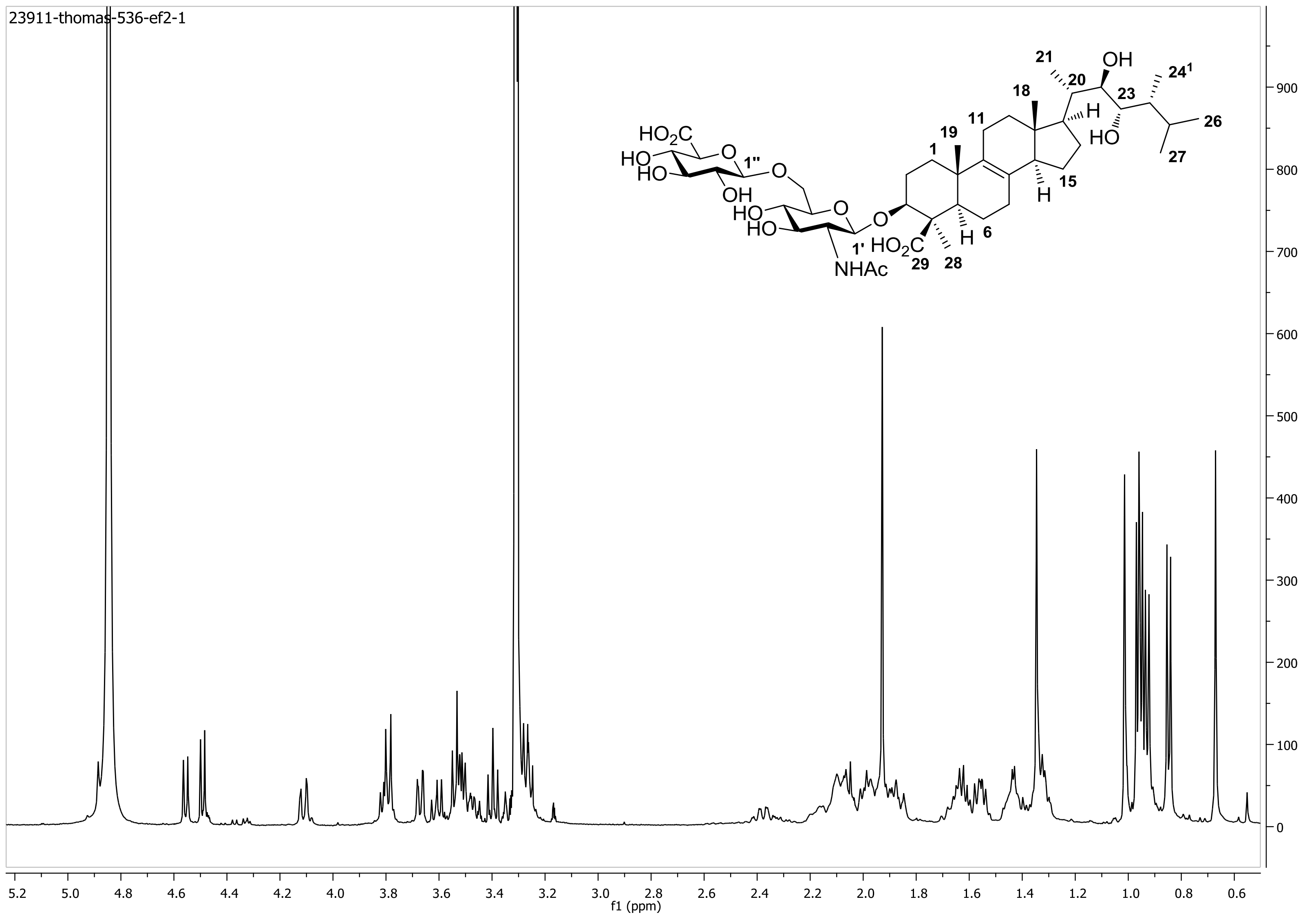

Figure S2. ^{13}C-NMR spectrum of **1** (125 MHz) in CD_3OD.

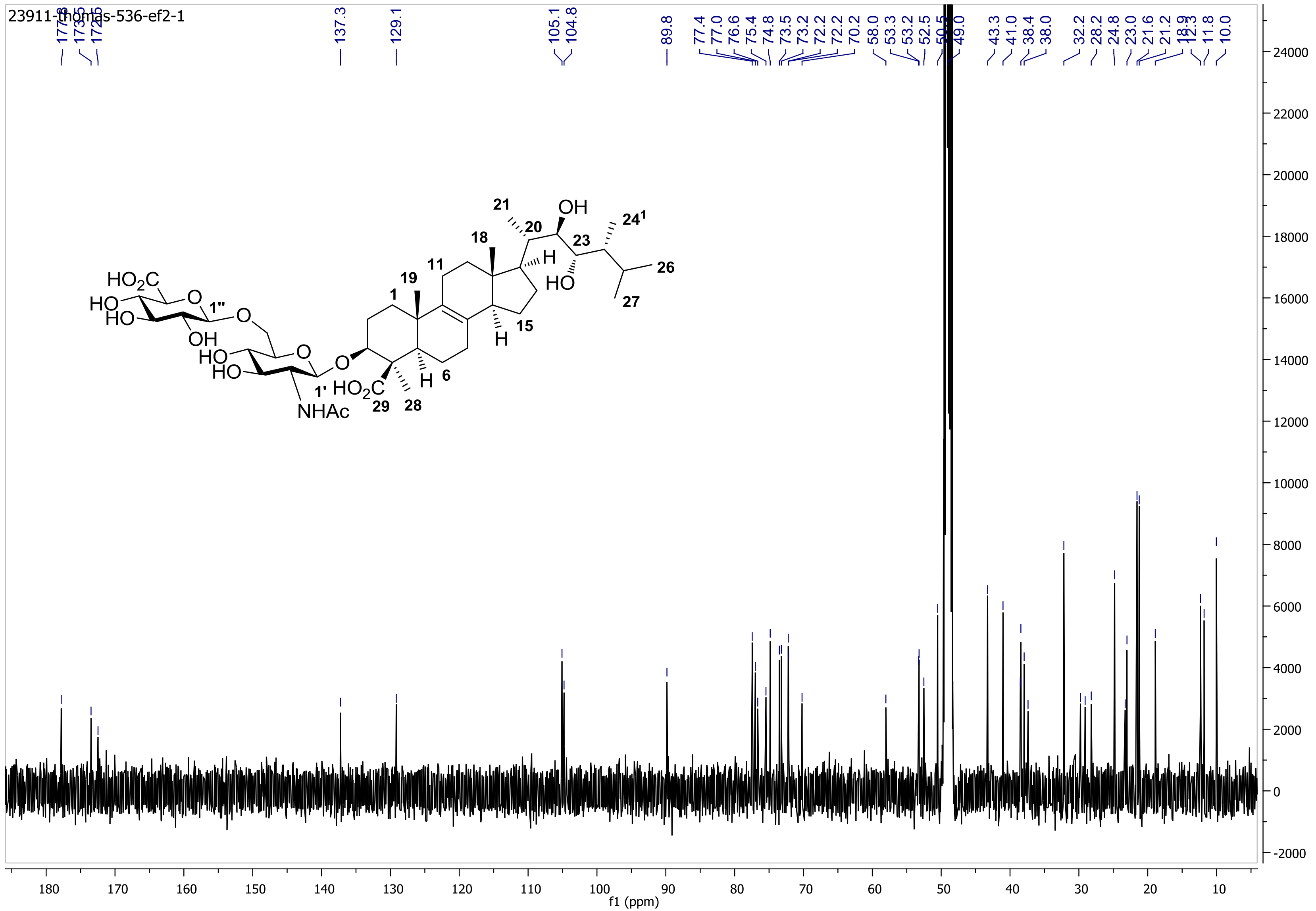

Figure S3. COSY spectrum of **1** (500 MHz) in CD_3OD.

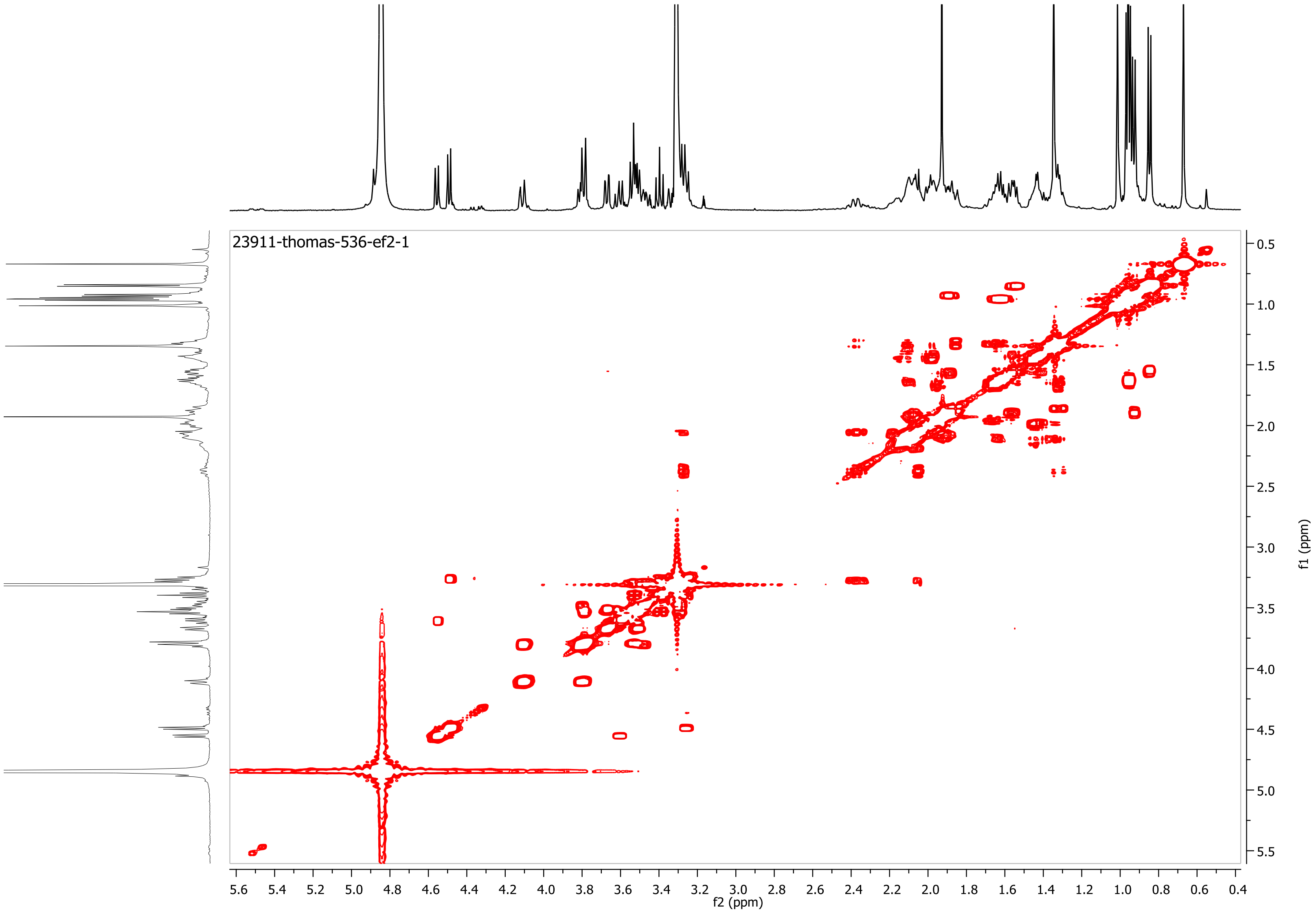

Figure S4. HSQC spectrum of **1** in CD_3OD (500 MHz).

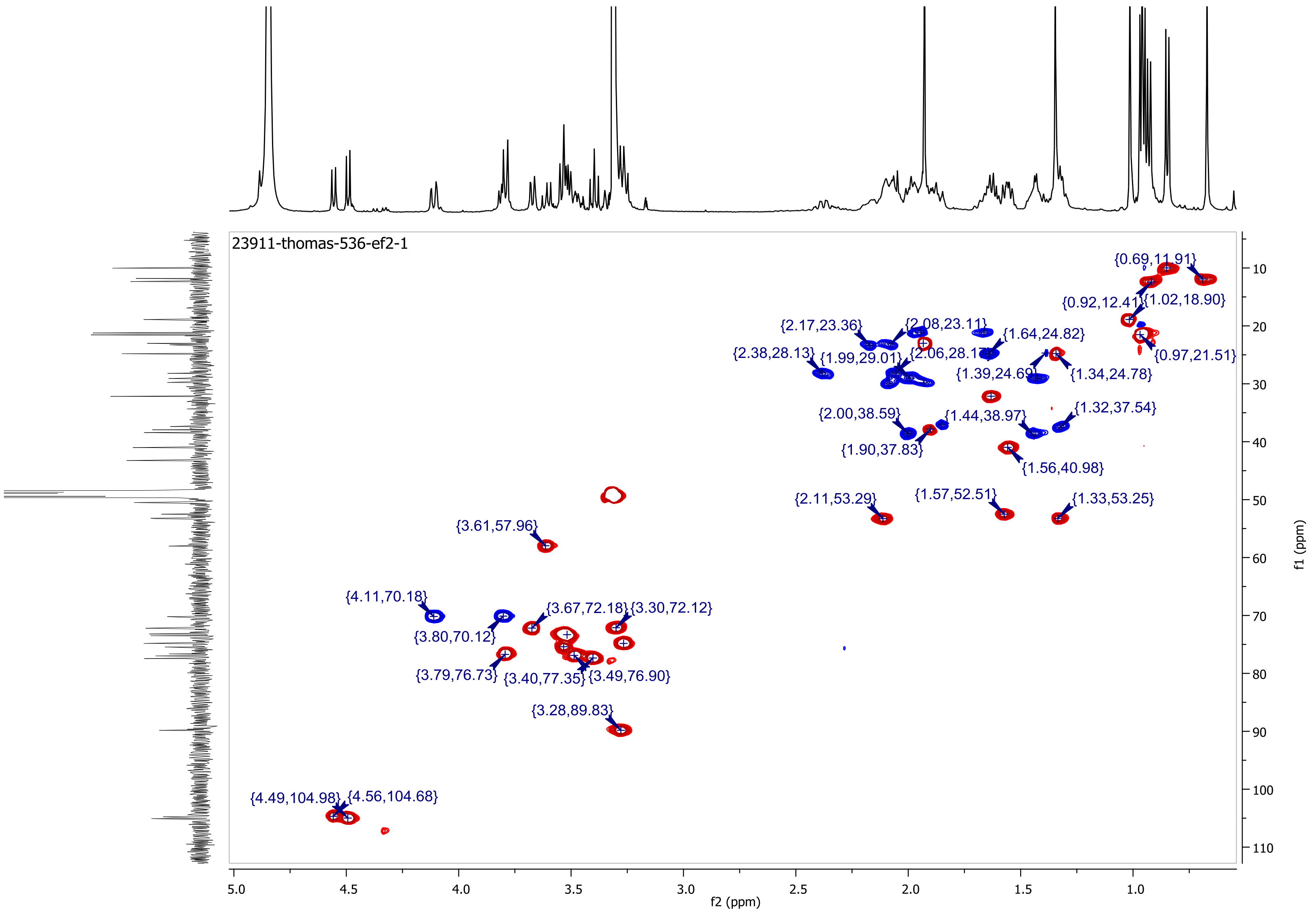

Figure S5. HMBC spectrum of **1** in CD_3OD (500 MHz).

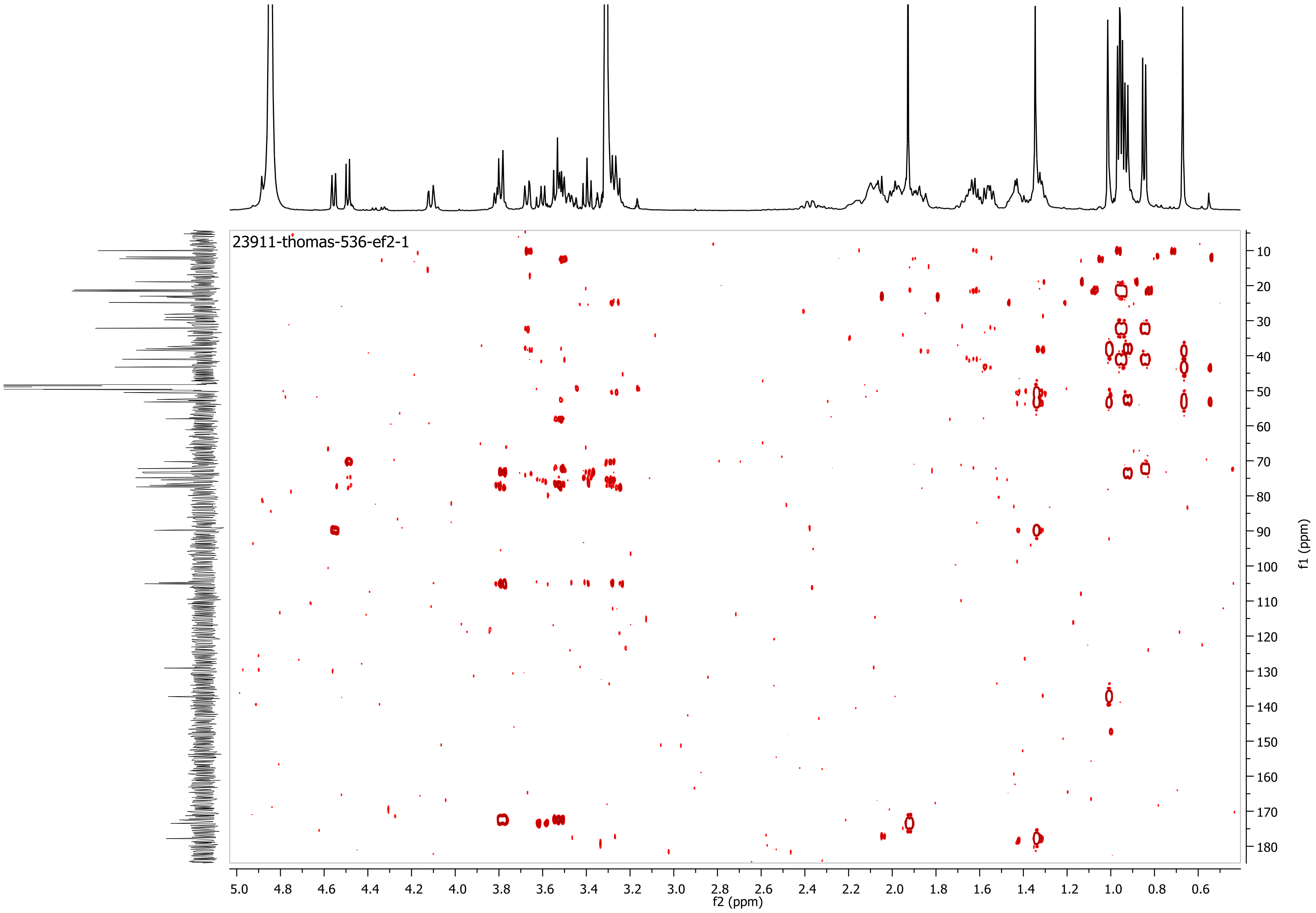

Figure S6. TOCSY spectrum of **1** in CD_3OD (500 MHz).

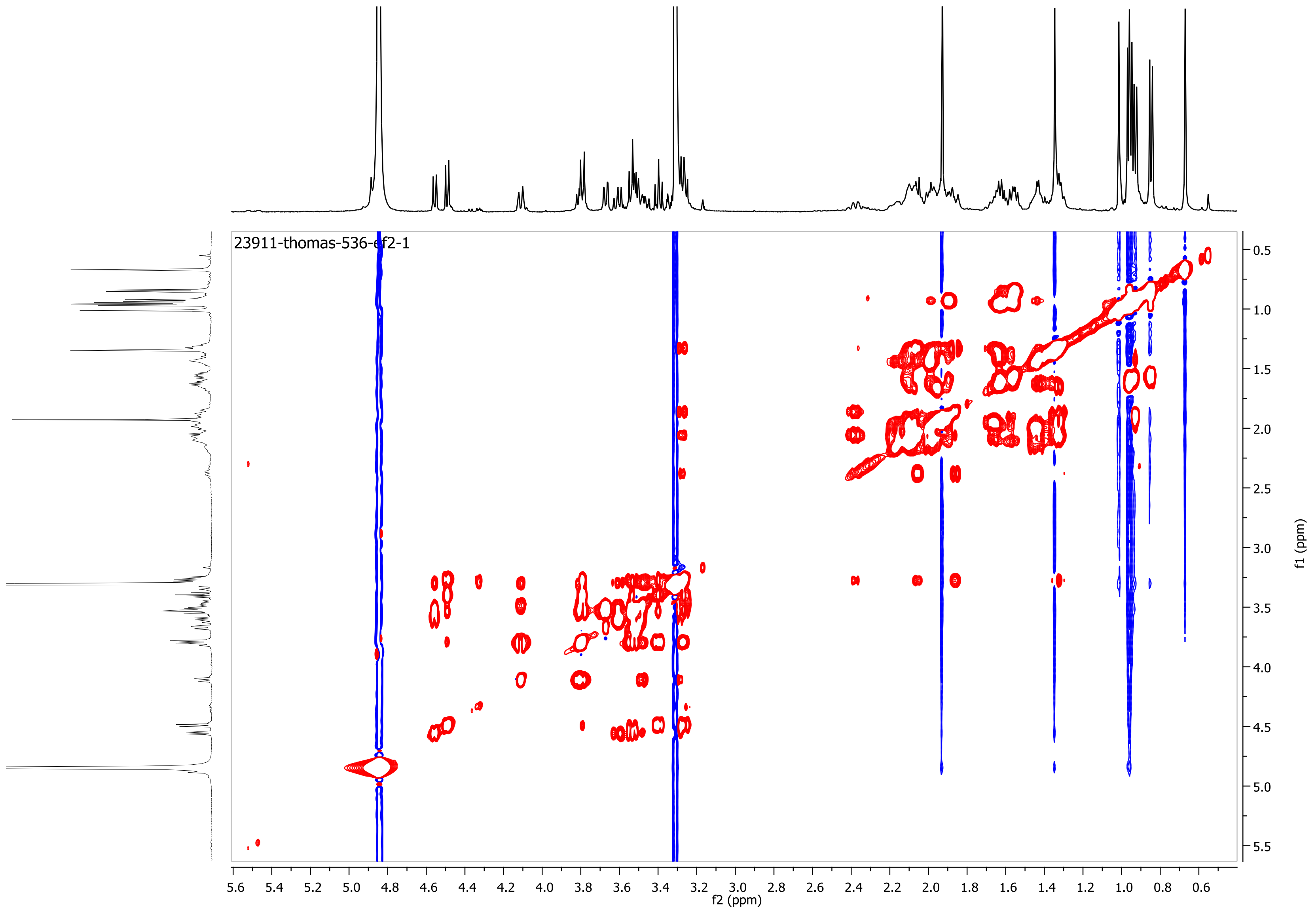

Figure S7. HRESIMS spectrum of **1.**

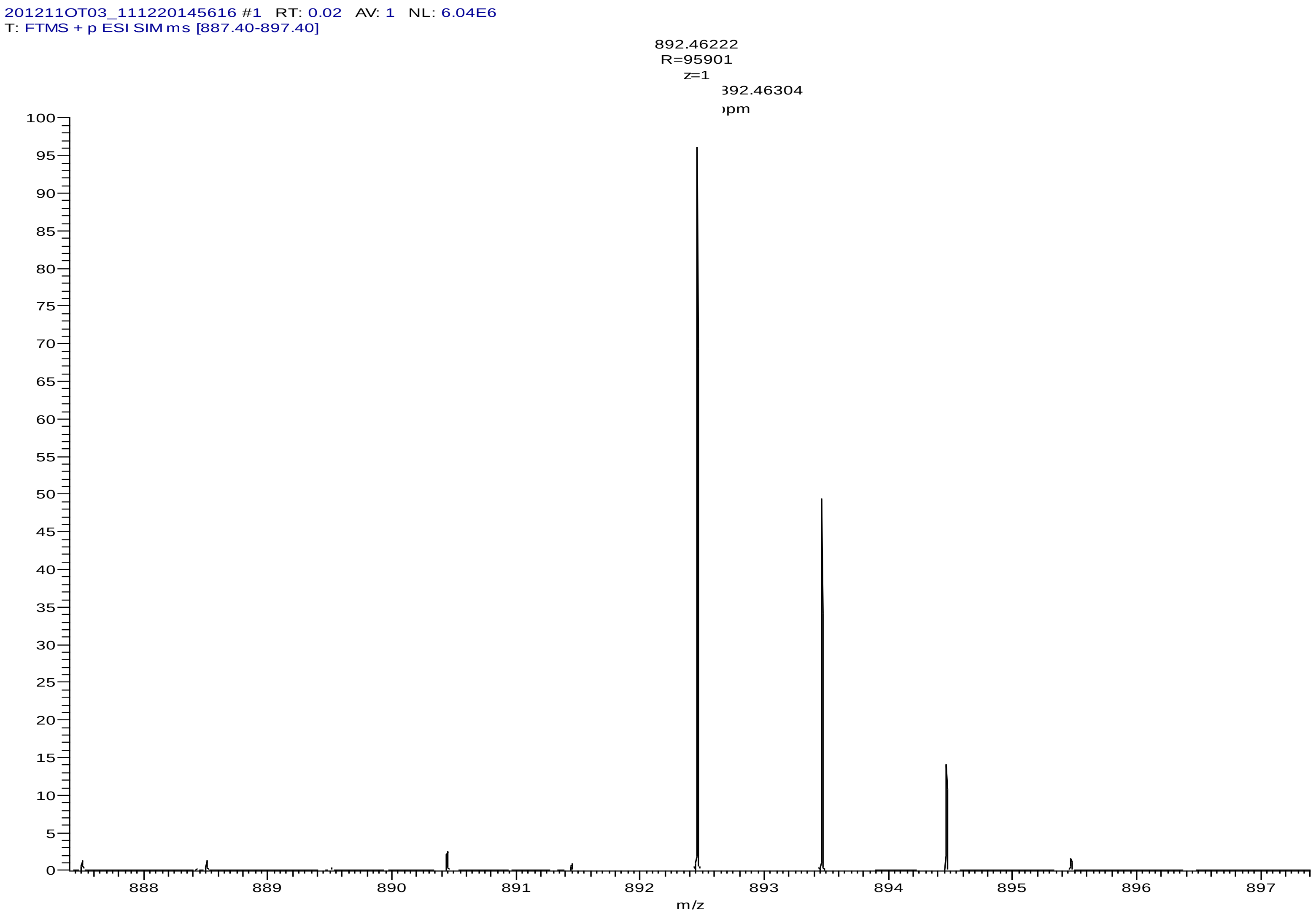

Figure S8. ^{1}H-NMR spectrum of **2** (500 MHz) in CD_3OD.

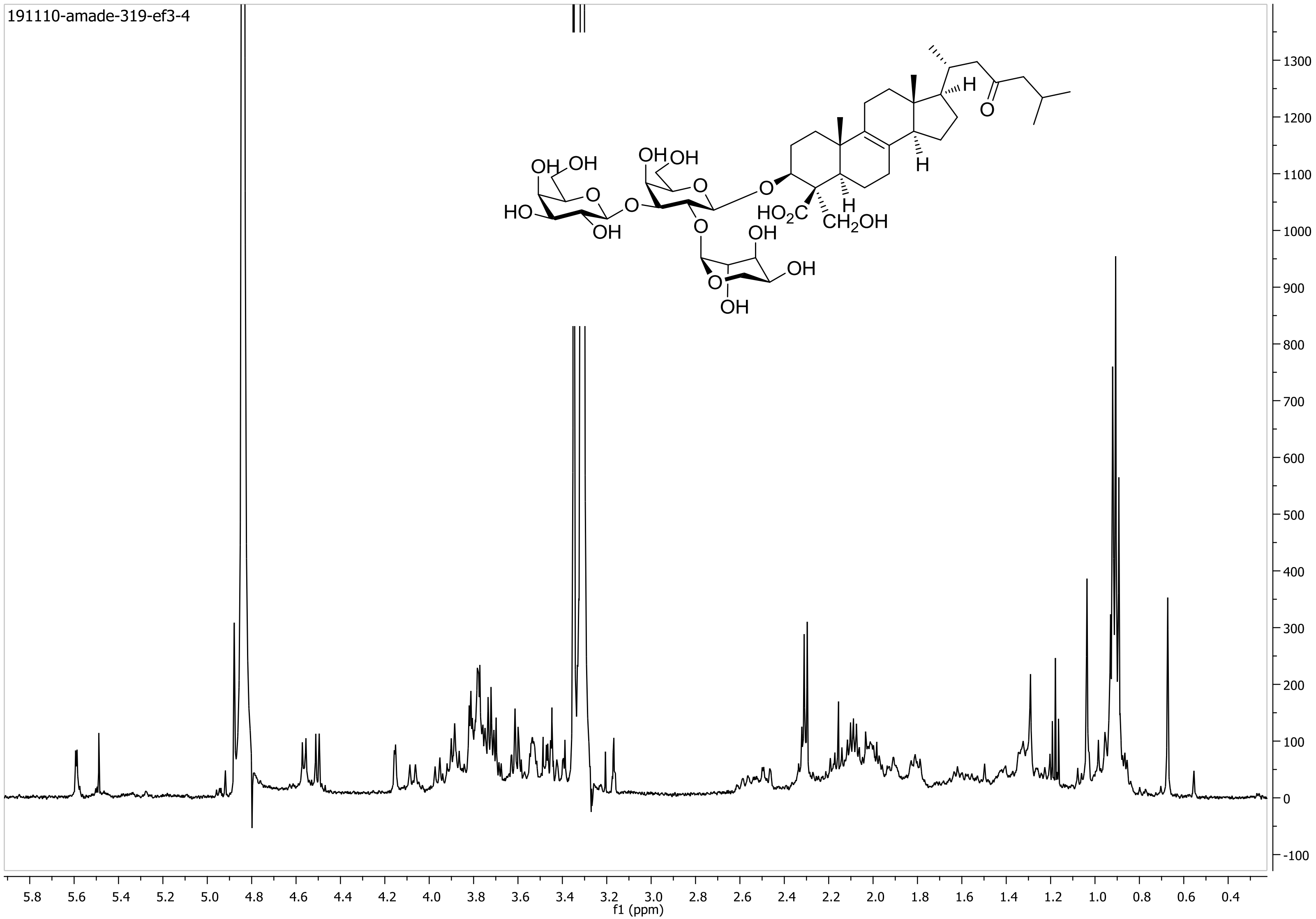

Figure S9. ^{13}C-NMR spectrum of **2** (125 MHz) in CD_3OD.

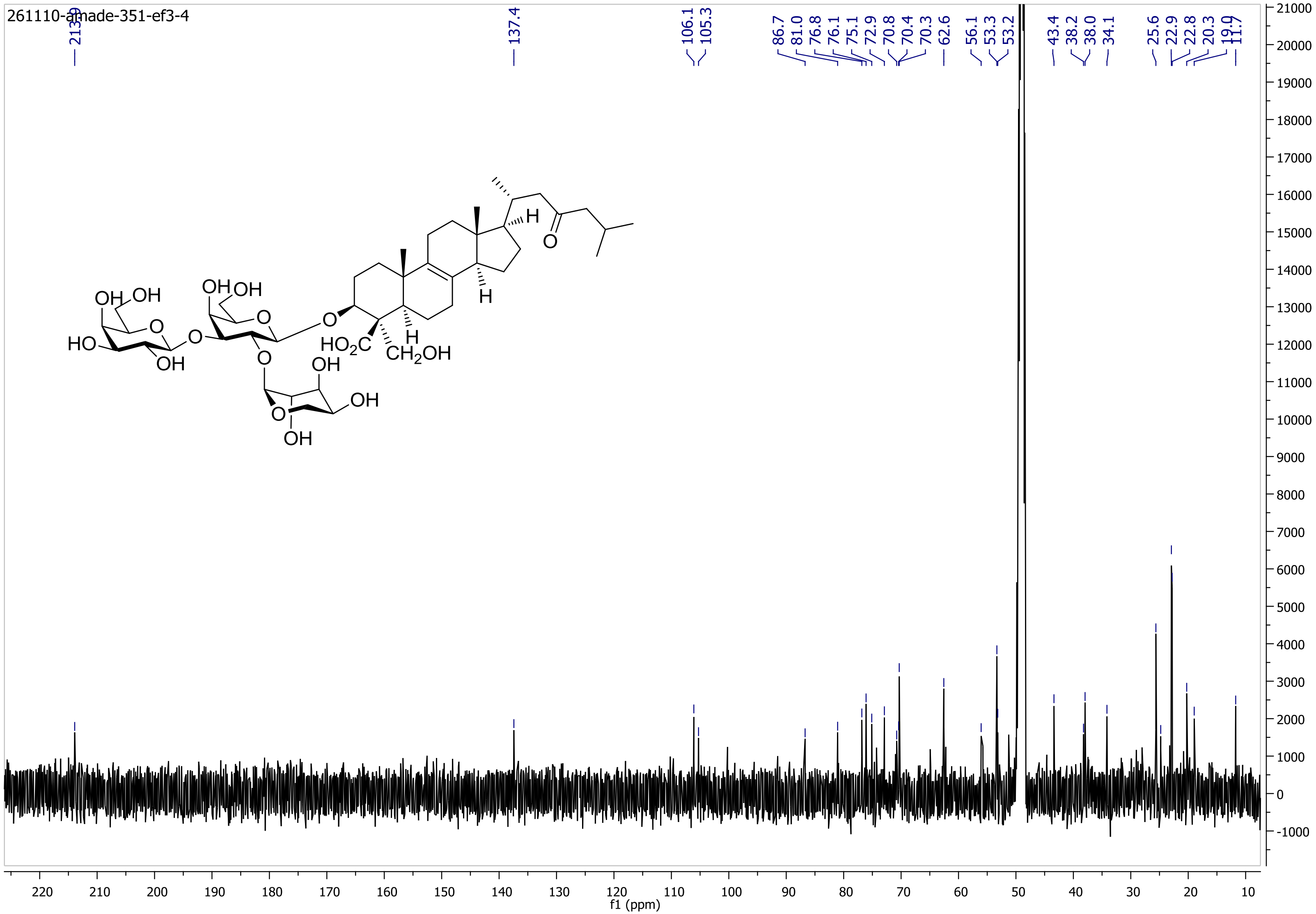

Figure S10. COSY spectrum of **2** (500 MHz) in CD_3OD.

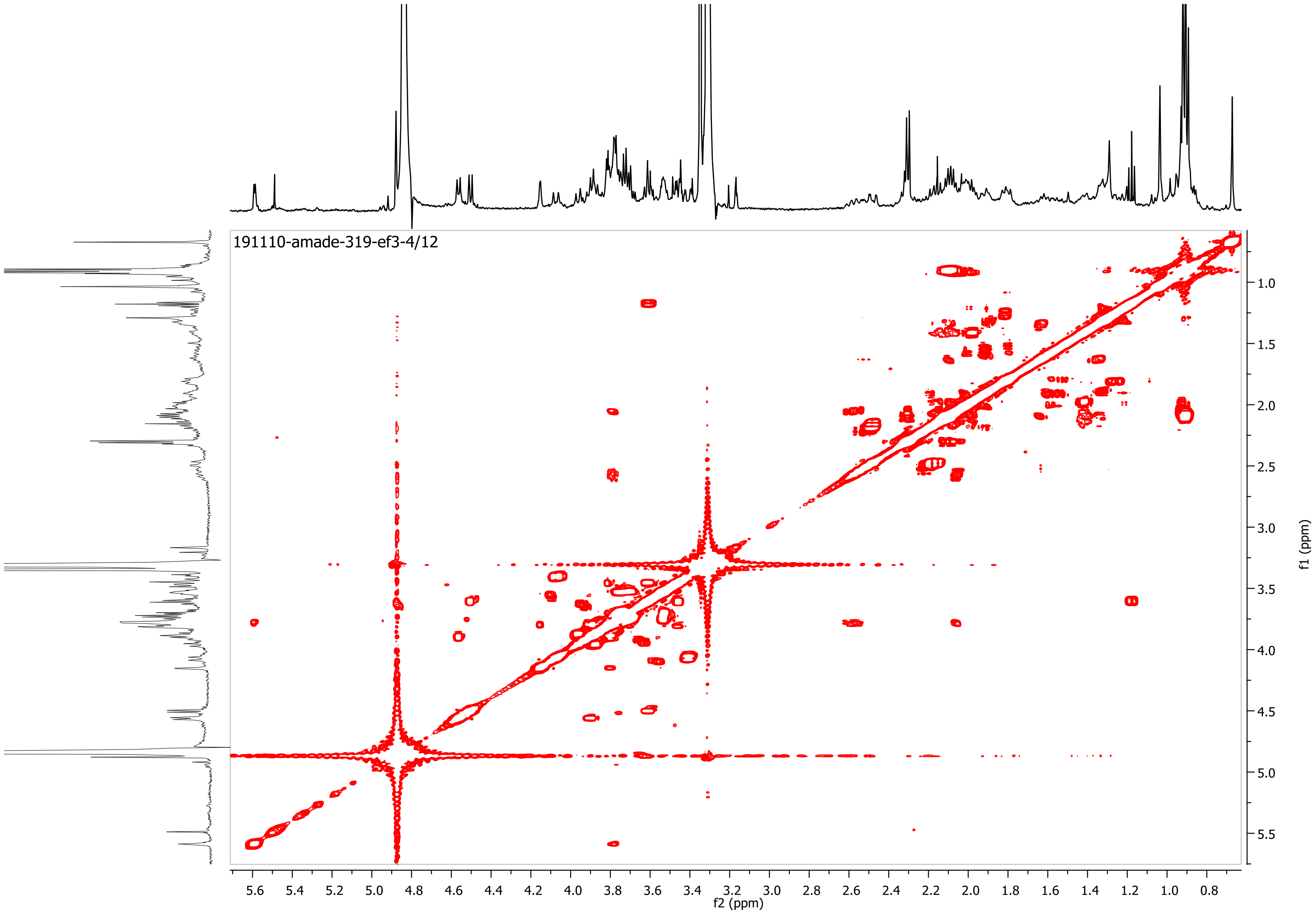

Figure S11. HSQC spectrum of **2** in CD_3OD.

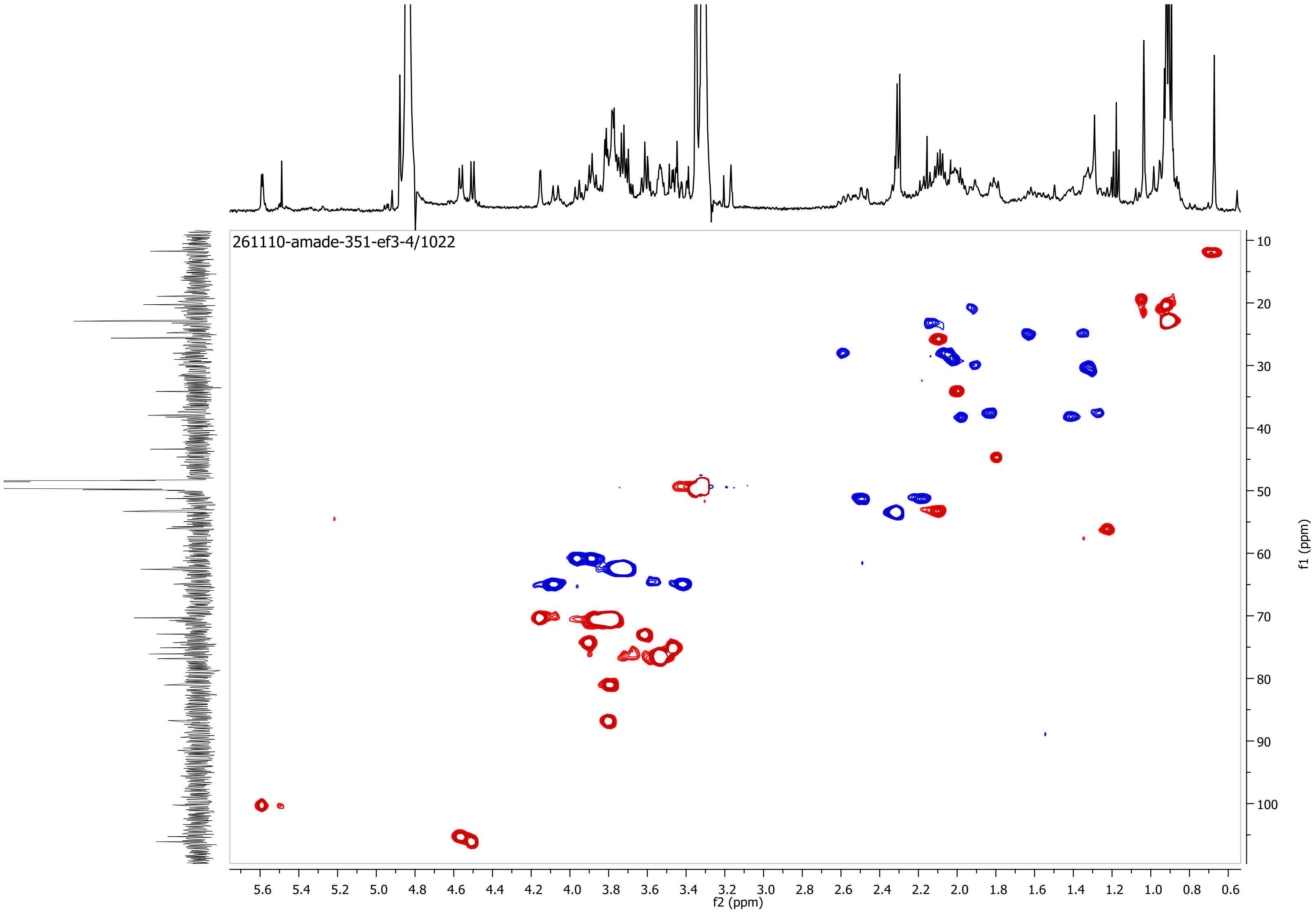

Figure S12. HMBC spectrum of **2** in CD_3OD.

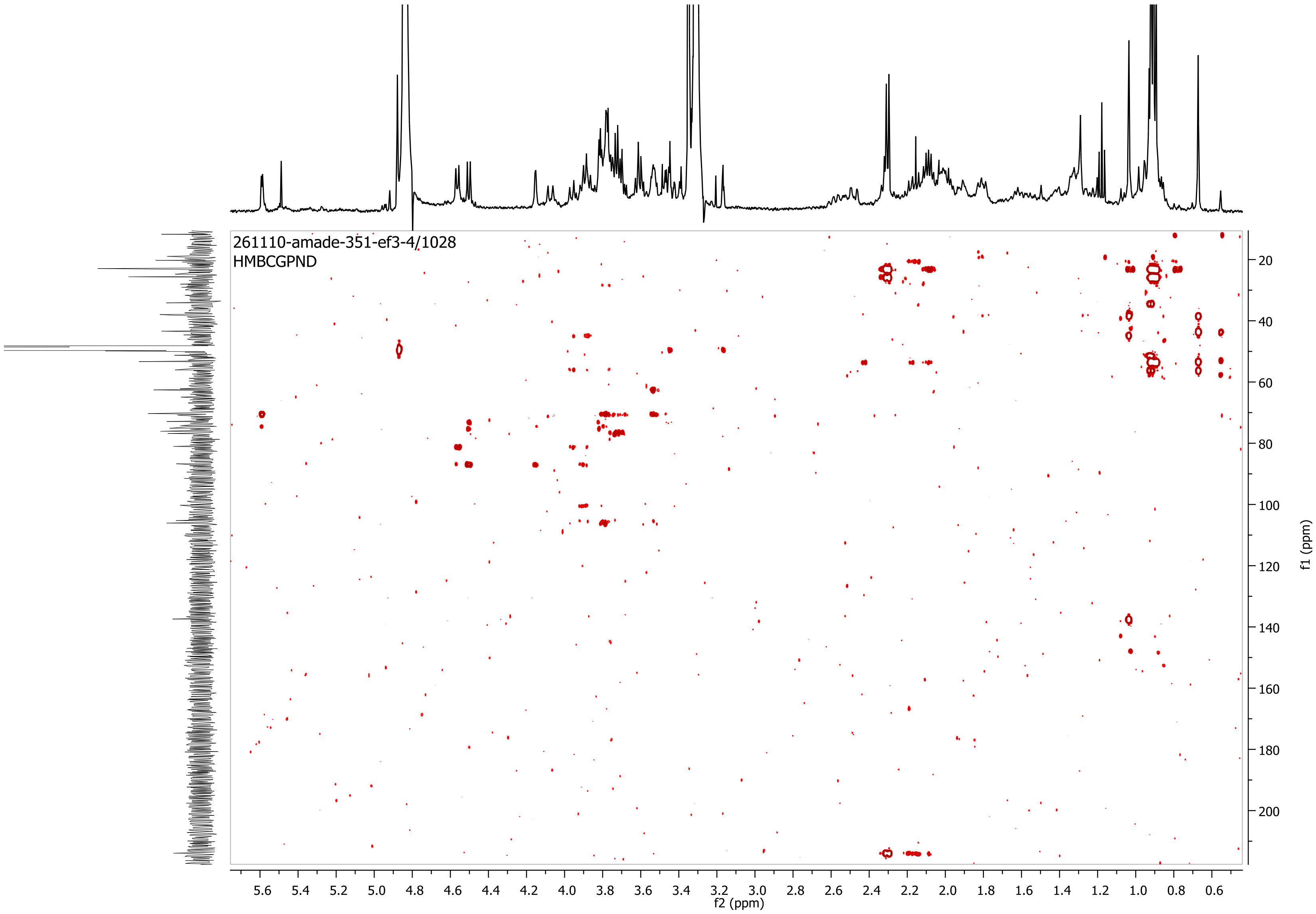

Figure S13. Zoom of the HMBC spectrum of **2** to evidence the C-29 carboxylic acid.

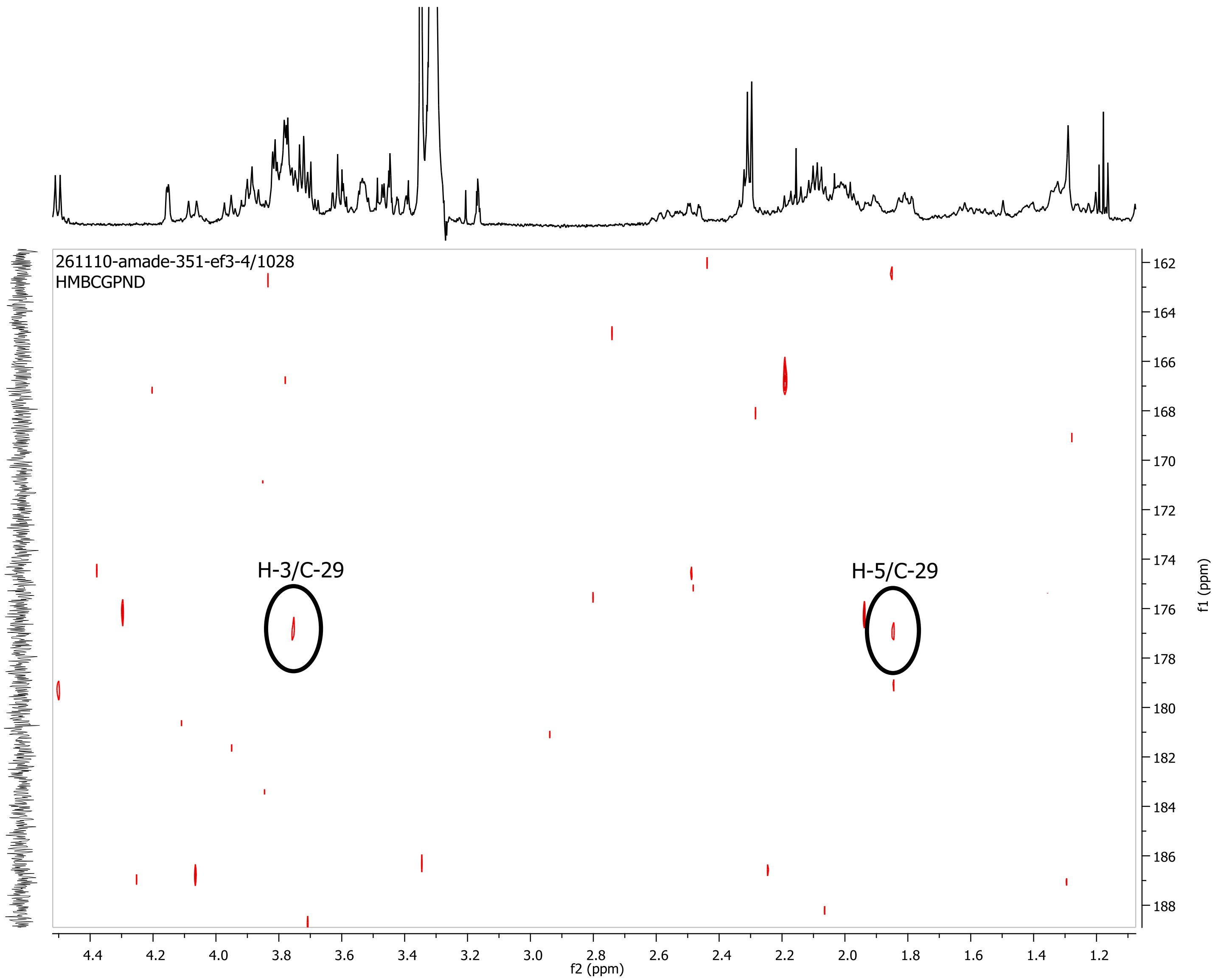

Figure S14. NOESY spectrum of **2** in CD_3OD.

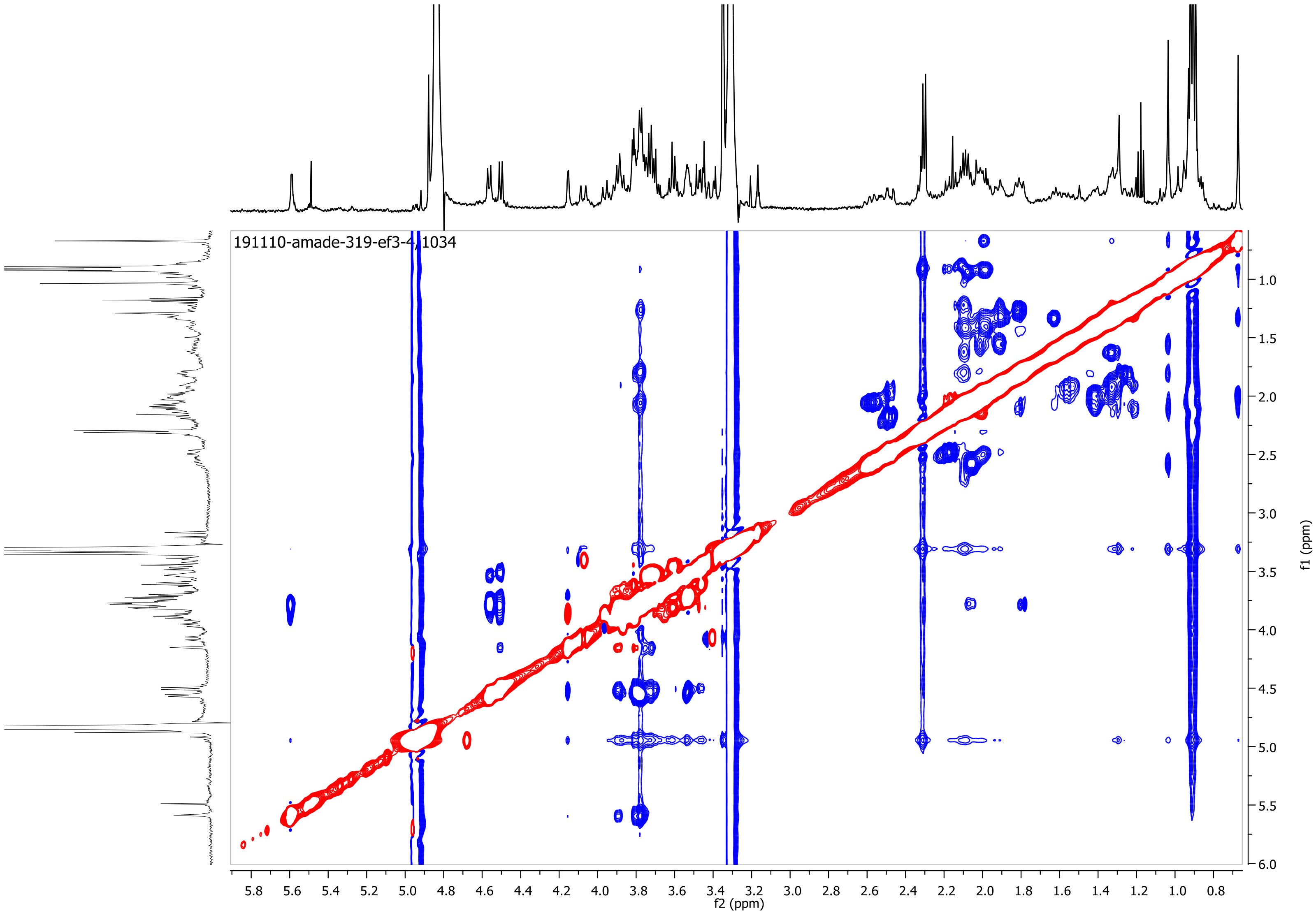

Figure S15. HRESIMS spectrum of **2.**

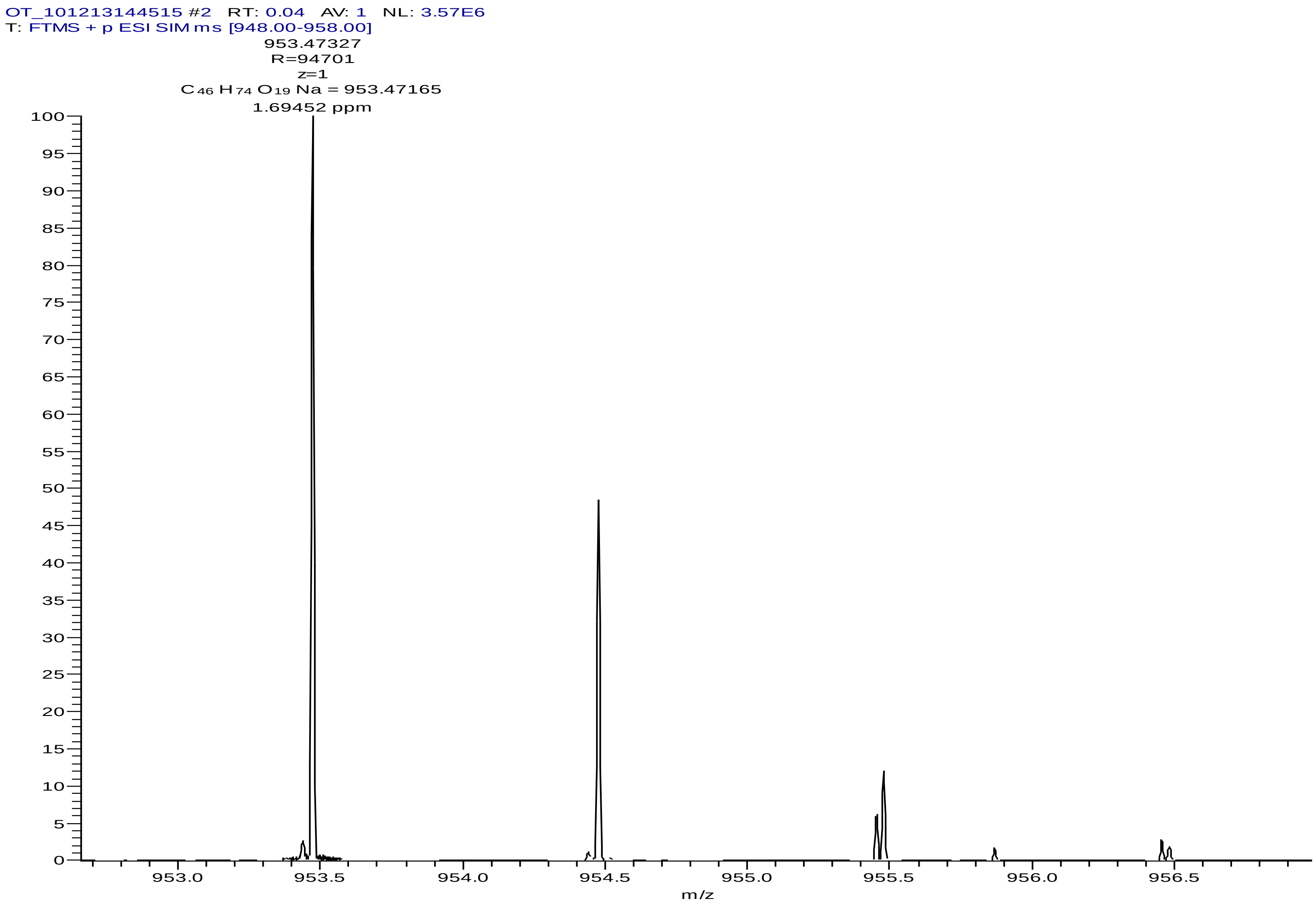

Figure S16. ^{1}H-NMR spectrum of **3** (500 MHz) in CD_3OD.

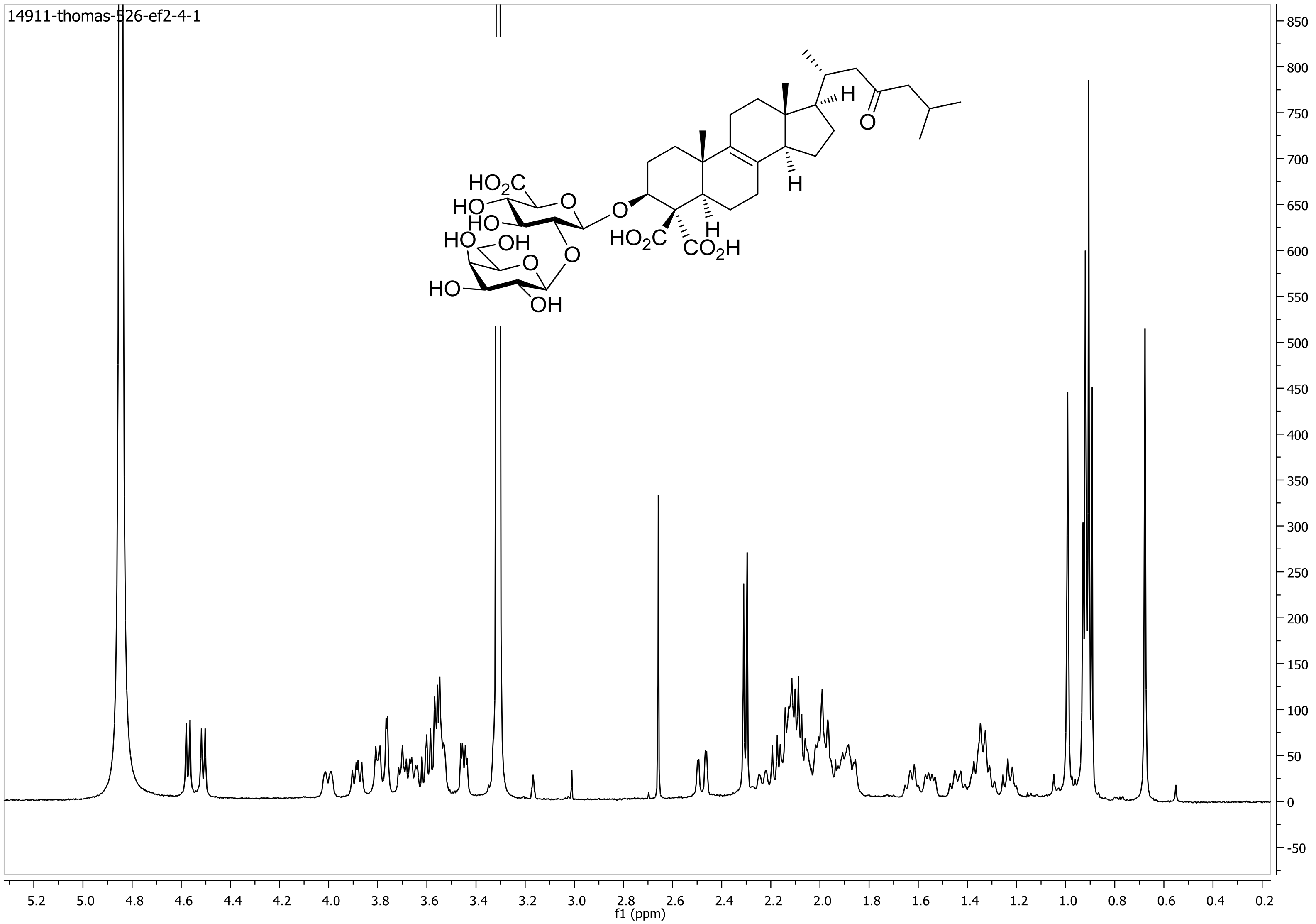

Figure S17. ^{13}C-NMR spectrum of **3** (125 MHz) in CD_3OD.

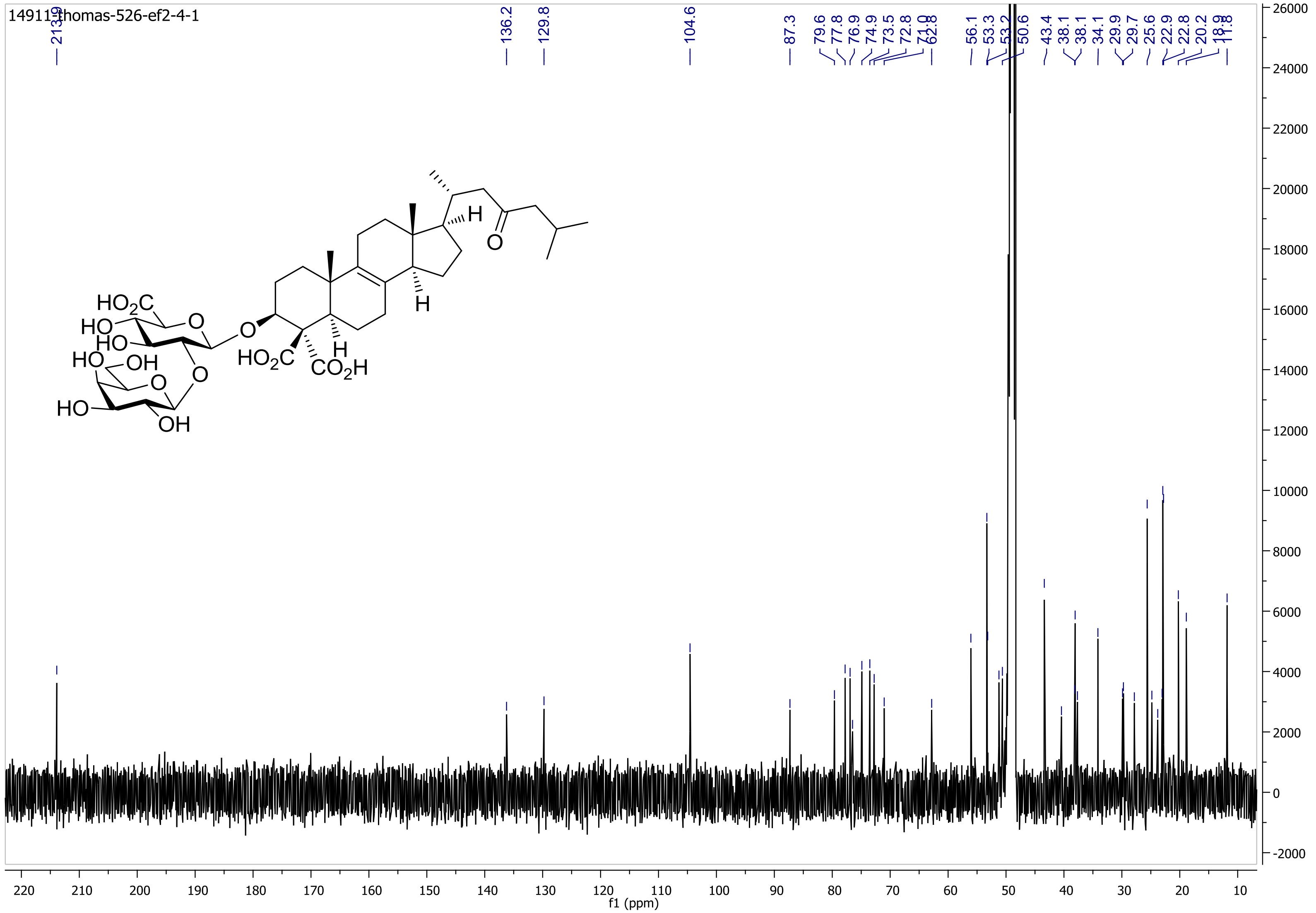

Figure S18. COSY spectrum of **3** (500 MHz) in CD_3OD.

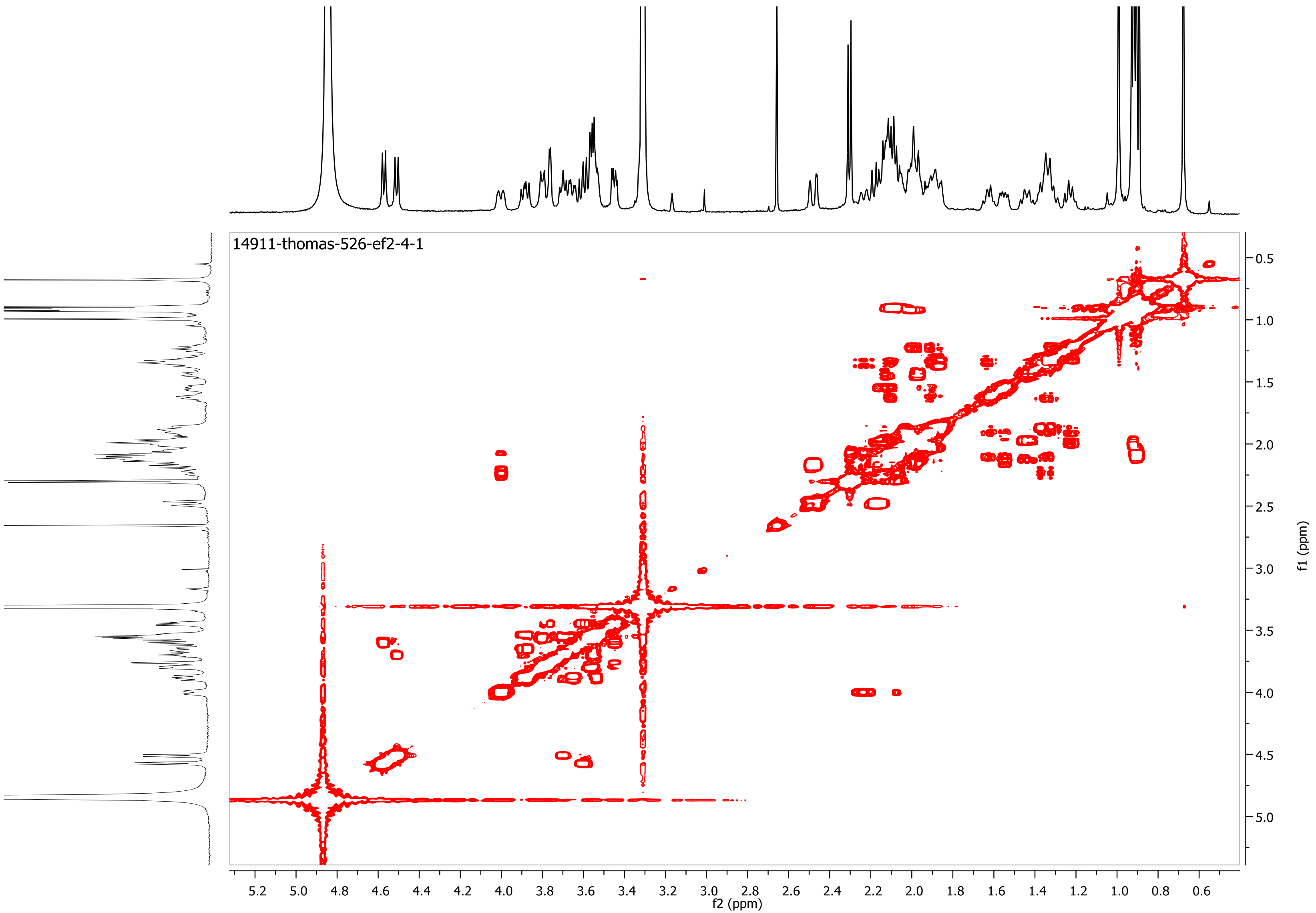

Figure S19. HSQC spectrum of **3** in CD_3OD.

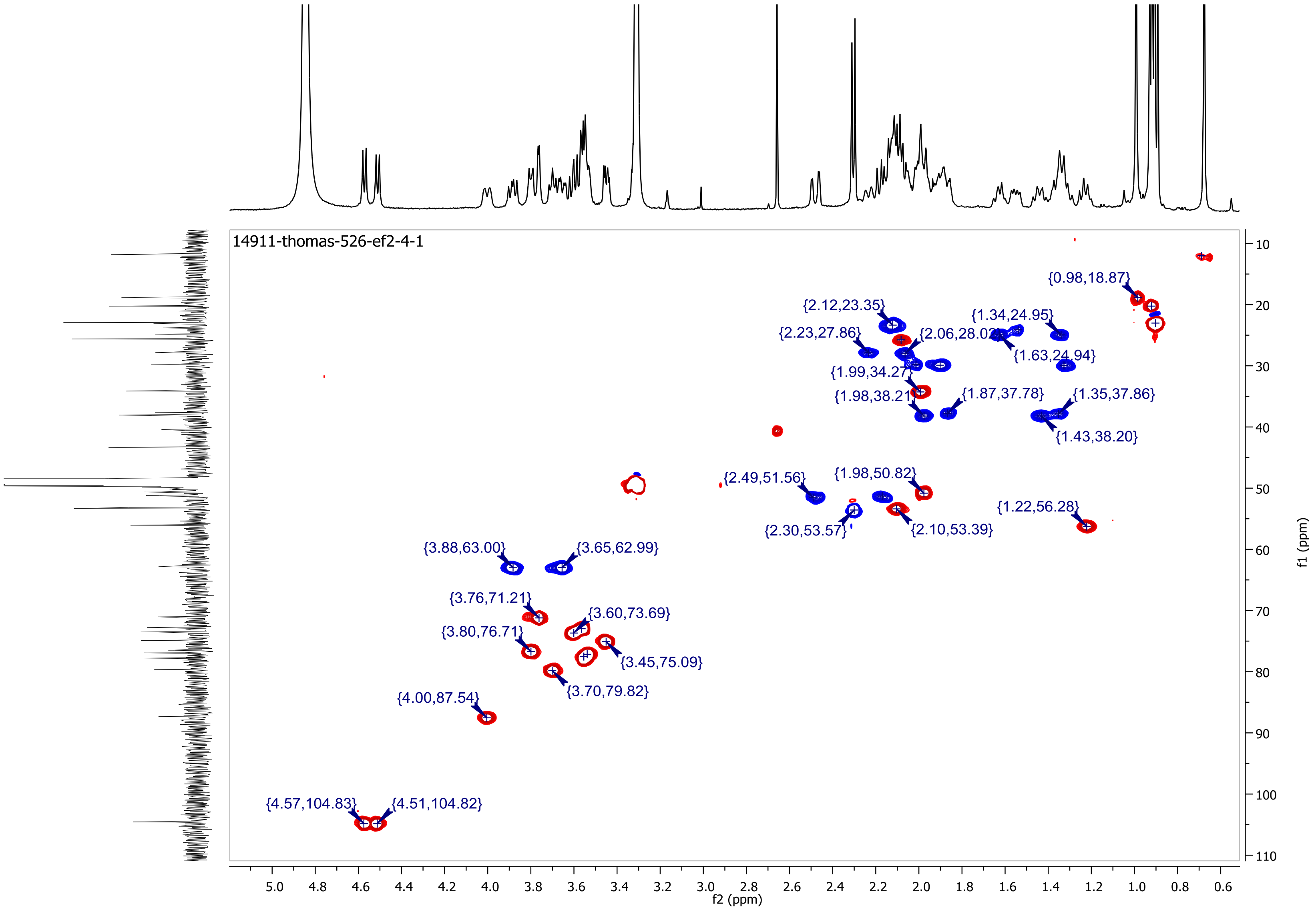

Figure S20. HMBC spectrum of **3** in CD_3OD.

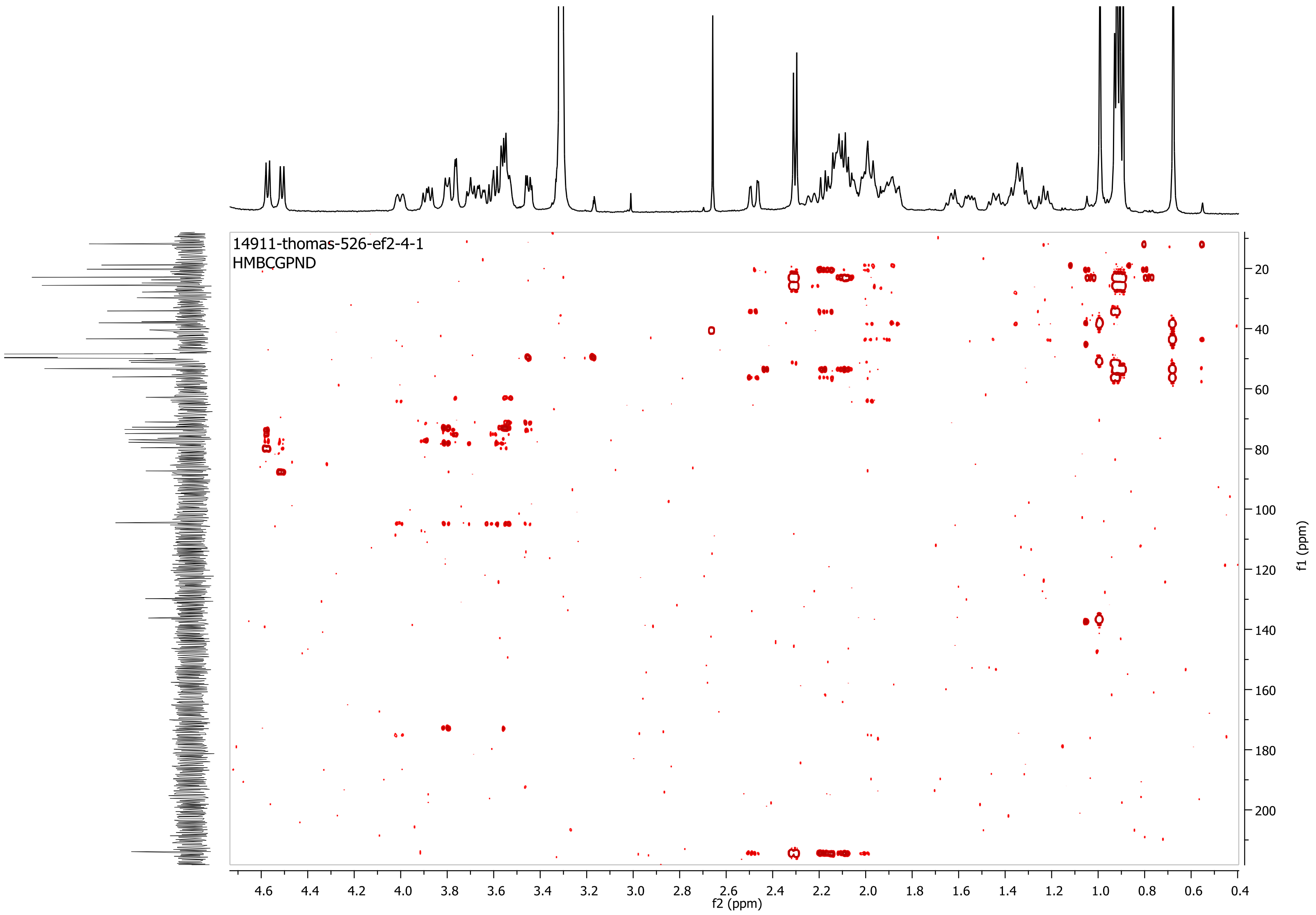

Figure S21. Zoom of the HMBC spectrum of **3**.

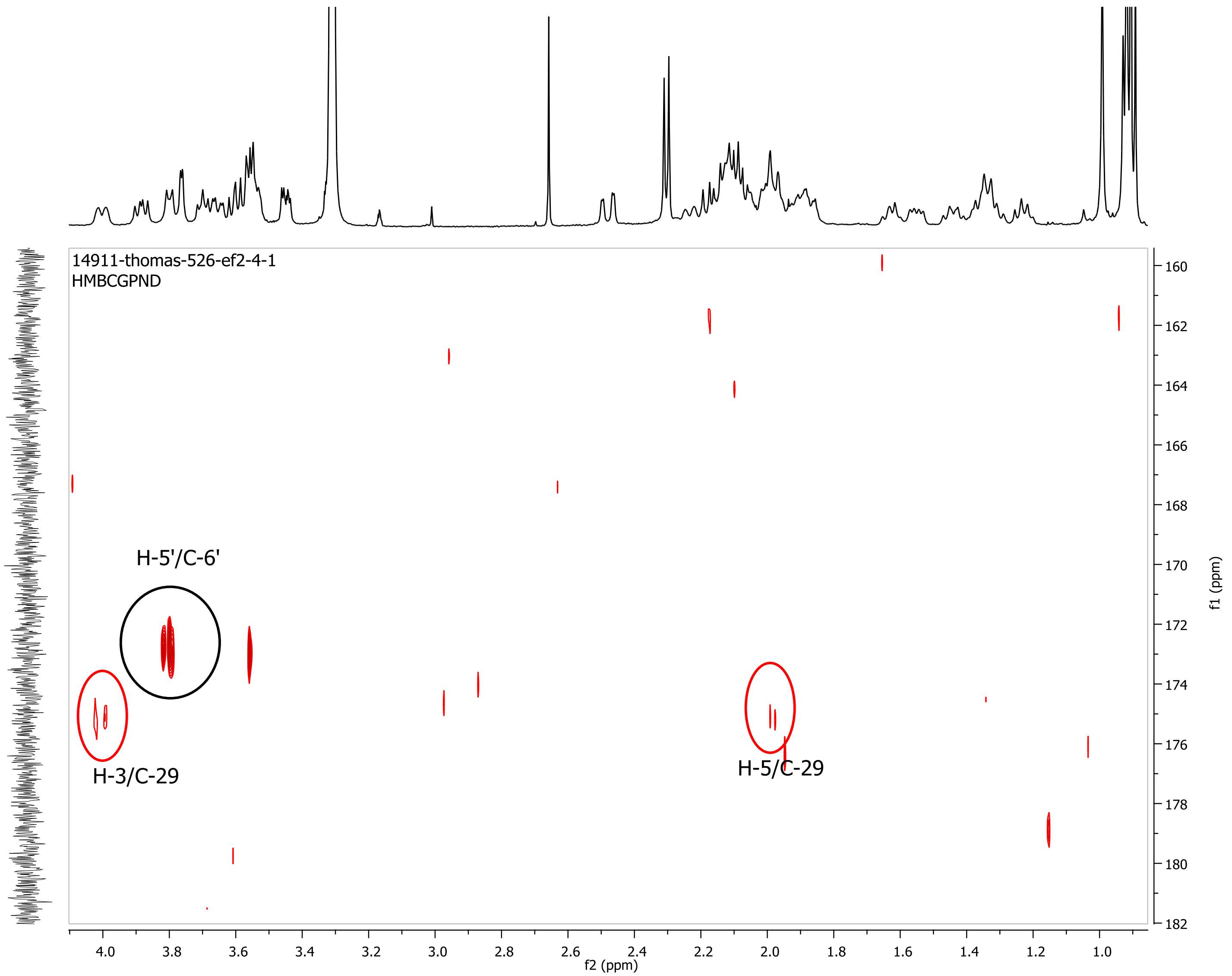

Figure S22. HRESIMS spectrum of **3.**

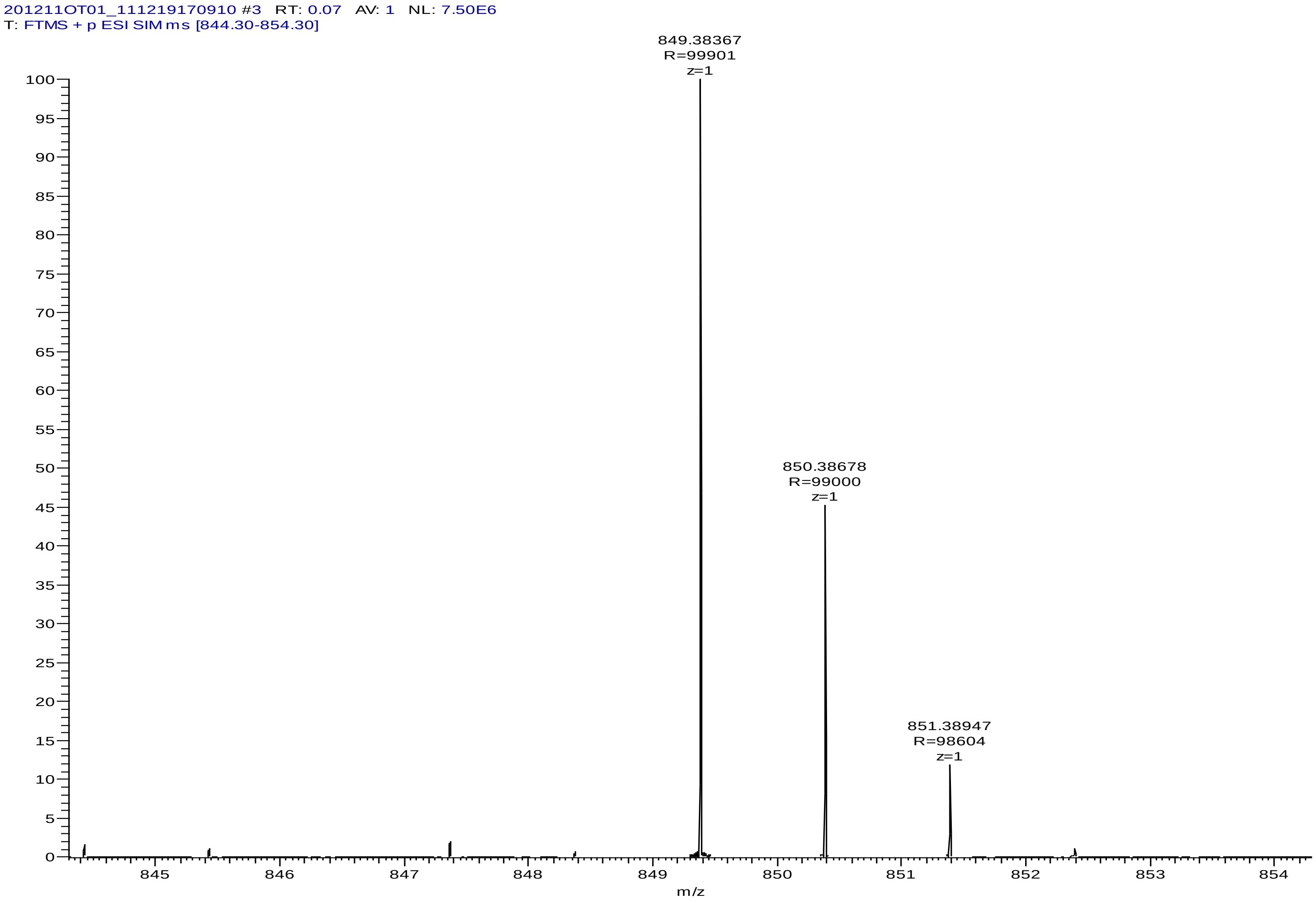

Figure S23. ^{1}H-NMR spectrum of **4** (500 MHz) in CD_3OD.

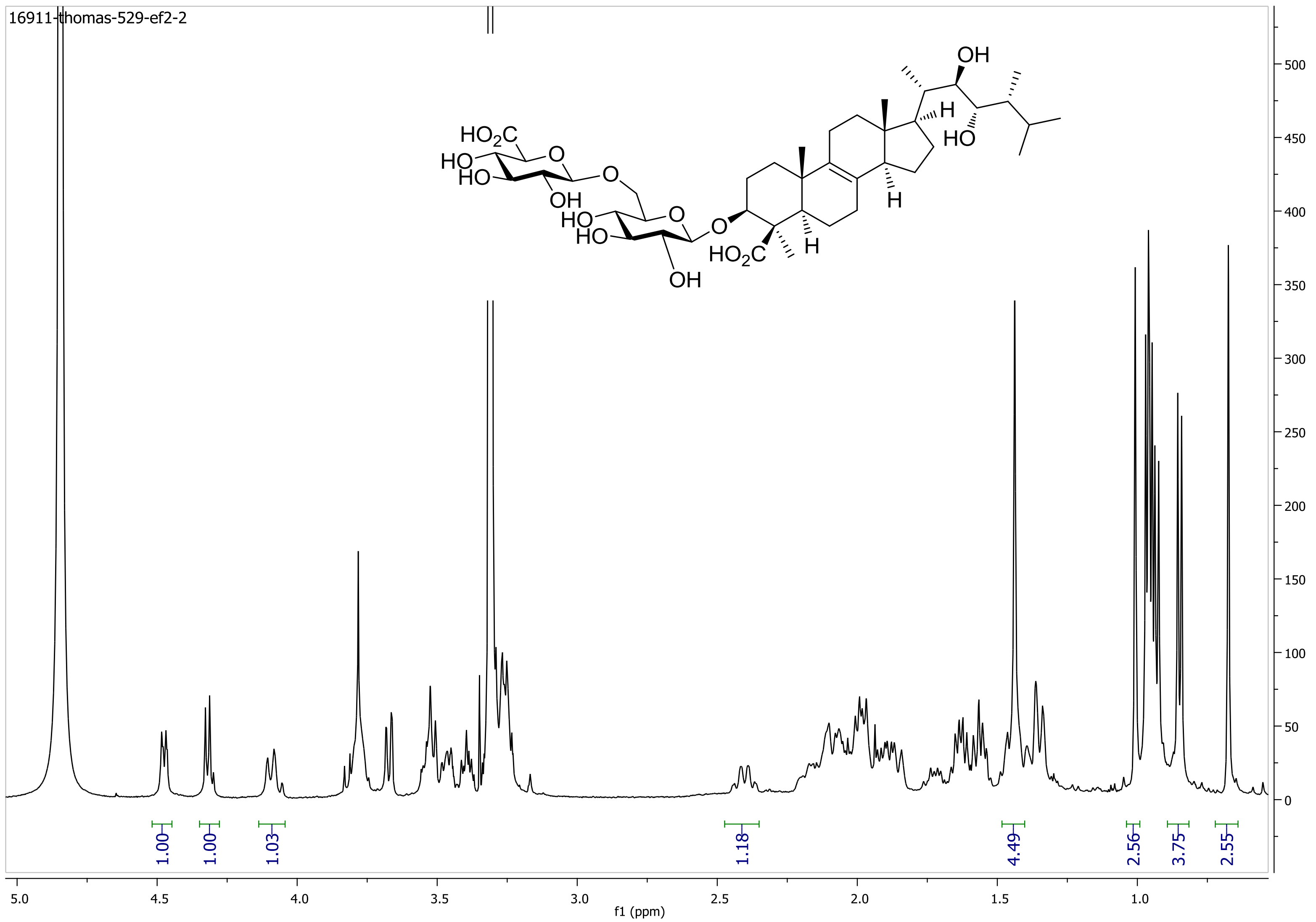

Figure S24. ^{13}C-NMR spectrum of **4** (125 MHz) in CD_3OD.

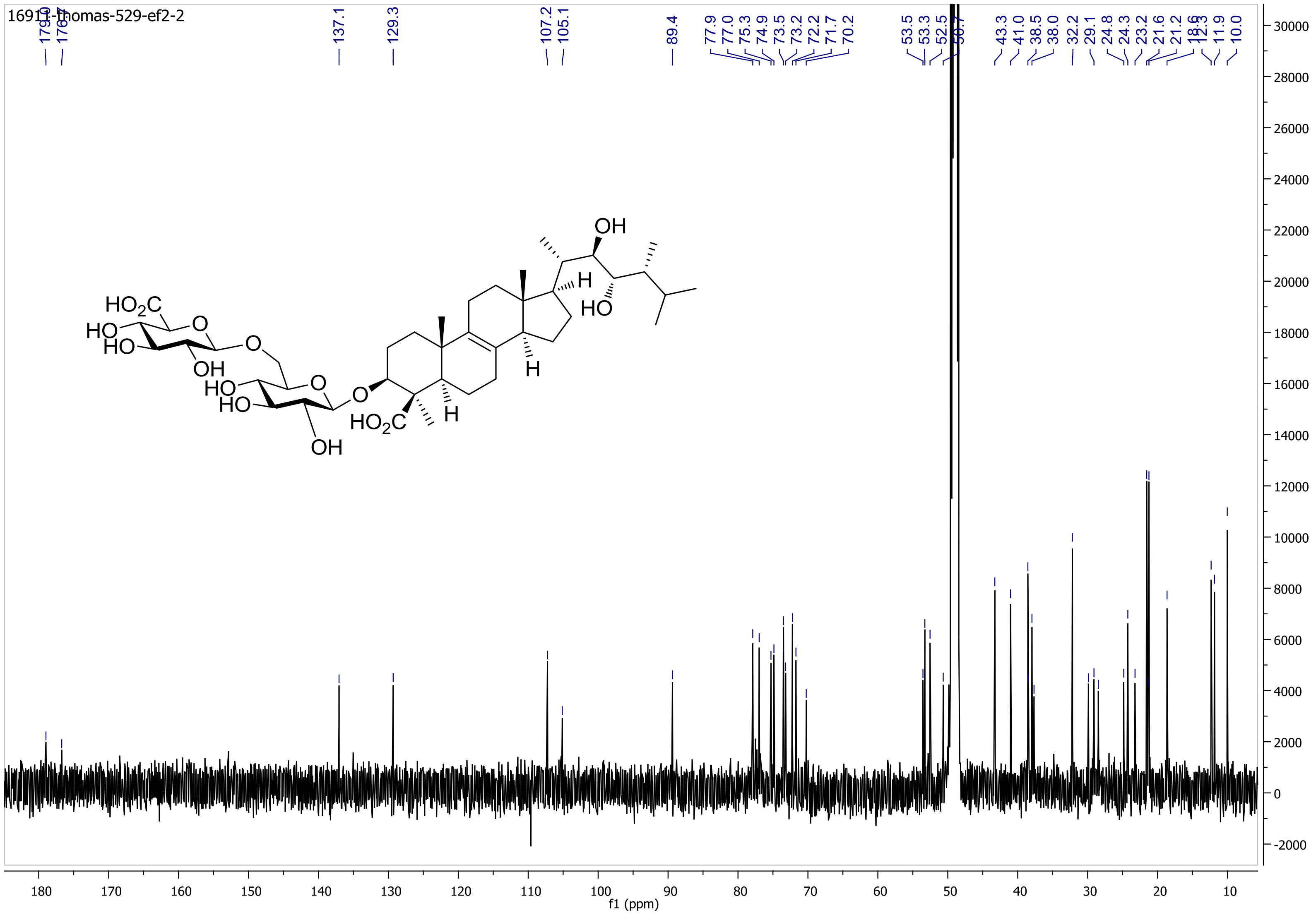

Figure S25. COSY spectrum of **4** (500 MHz) in CD_3OD.

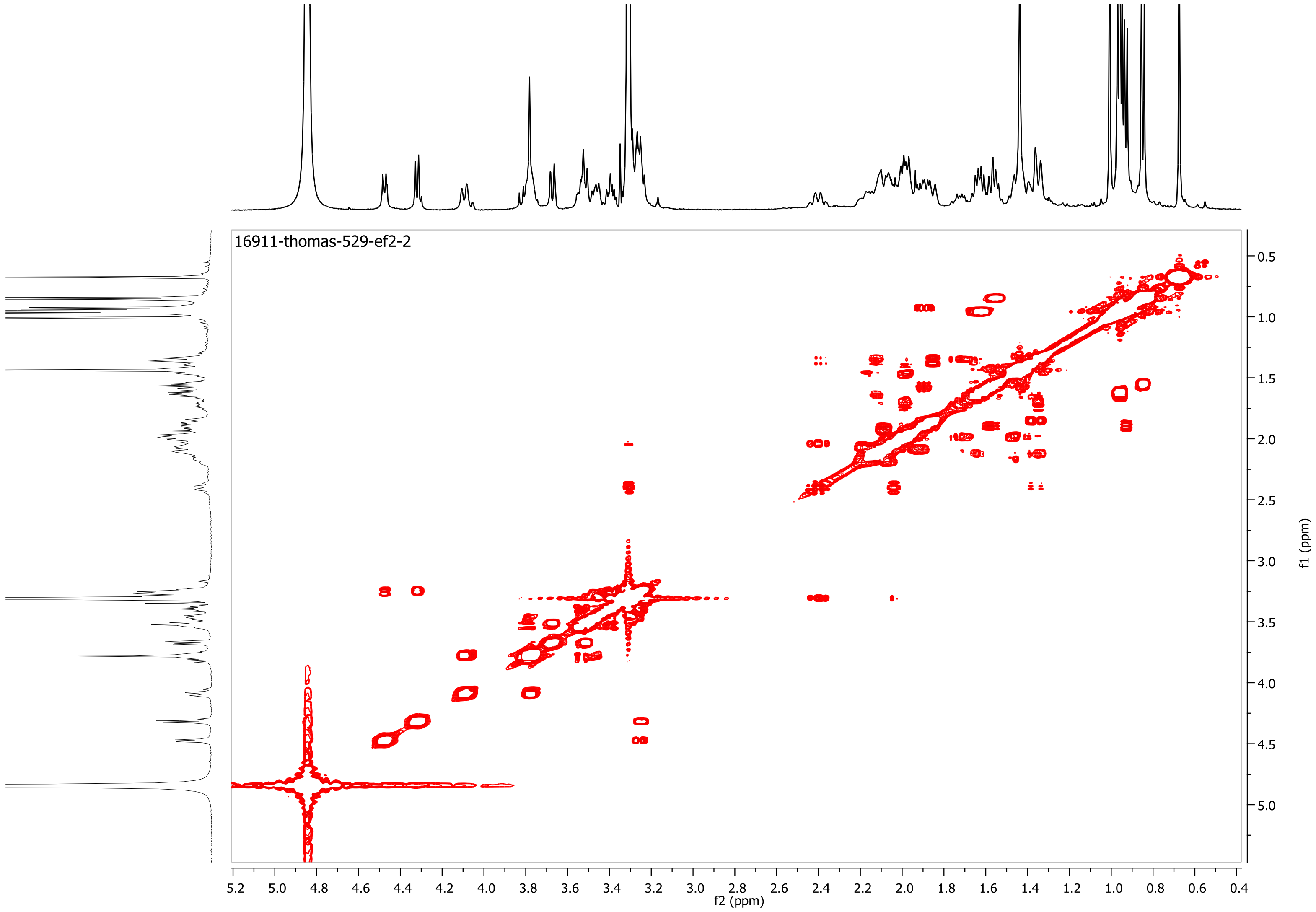

Figure S26. HSQC spectrum of **4** in CD_3OD.

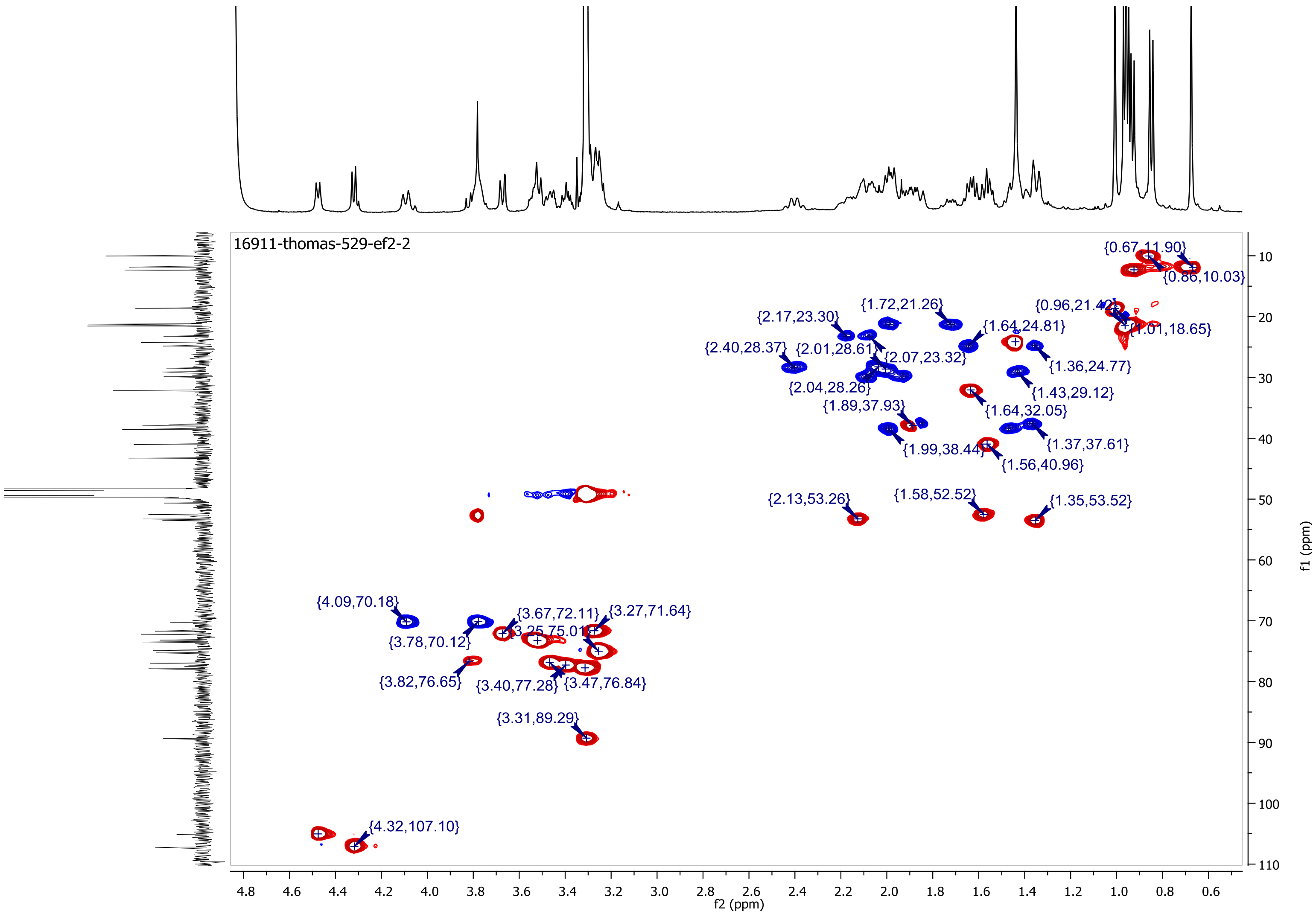

Figure S27. HMBC spectrum of **4** in CD_3OD.

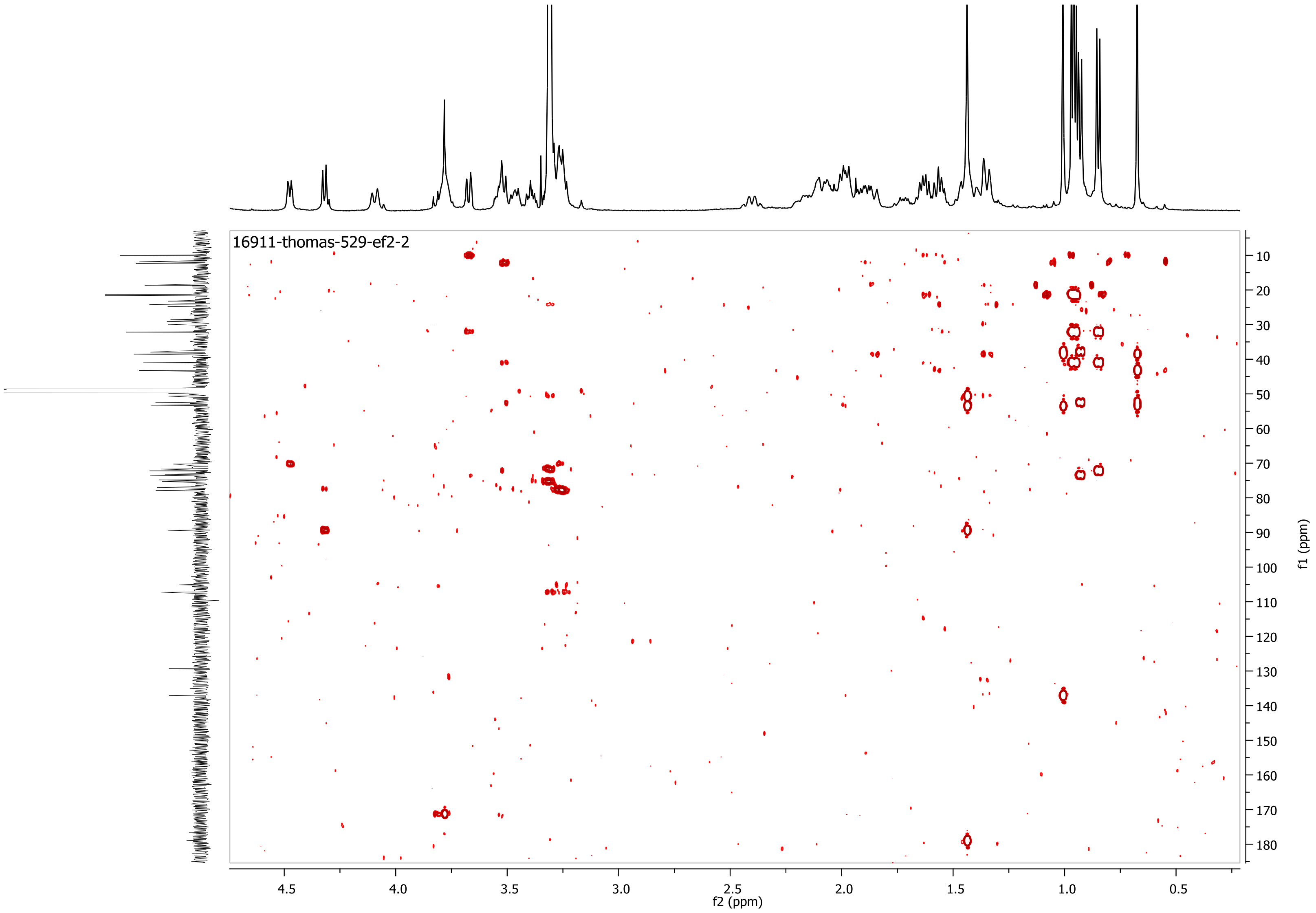

Figure S28. TOCSY spectrum of **4** in CD_3OD (500 MHz).

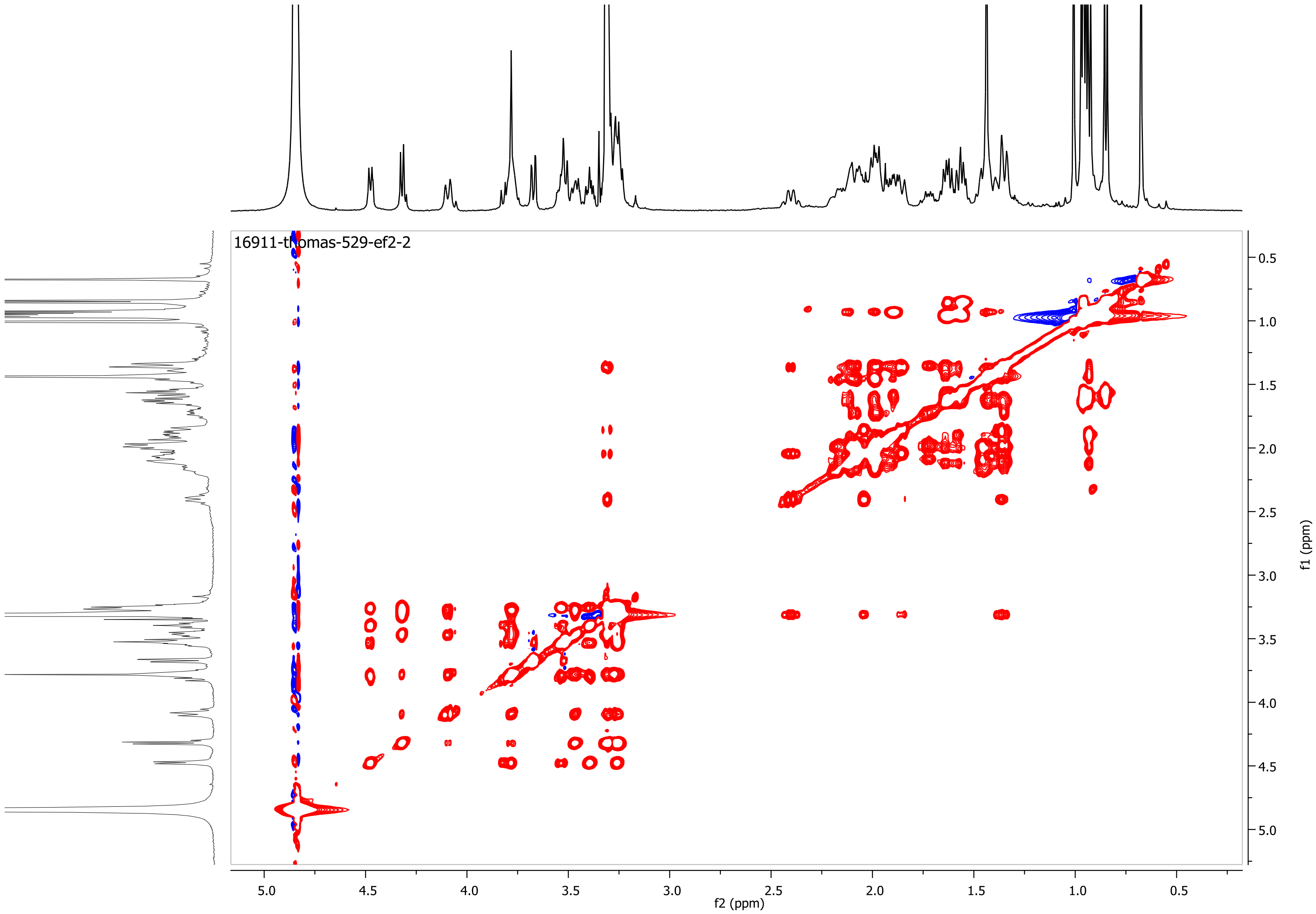

Figure S29. HRESIMS spectrum of **4.**

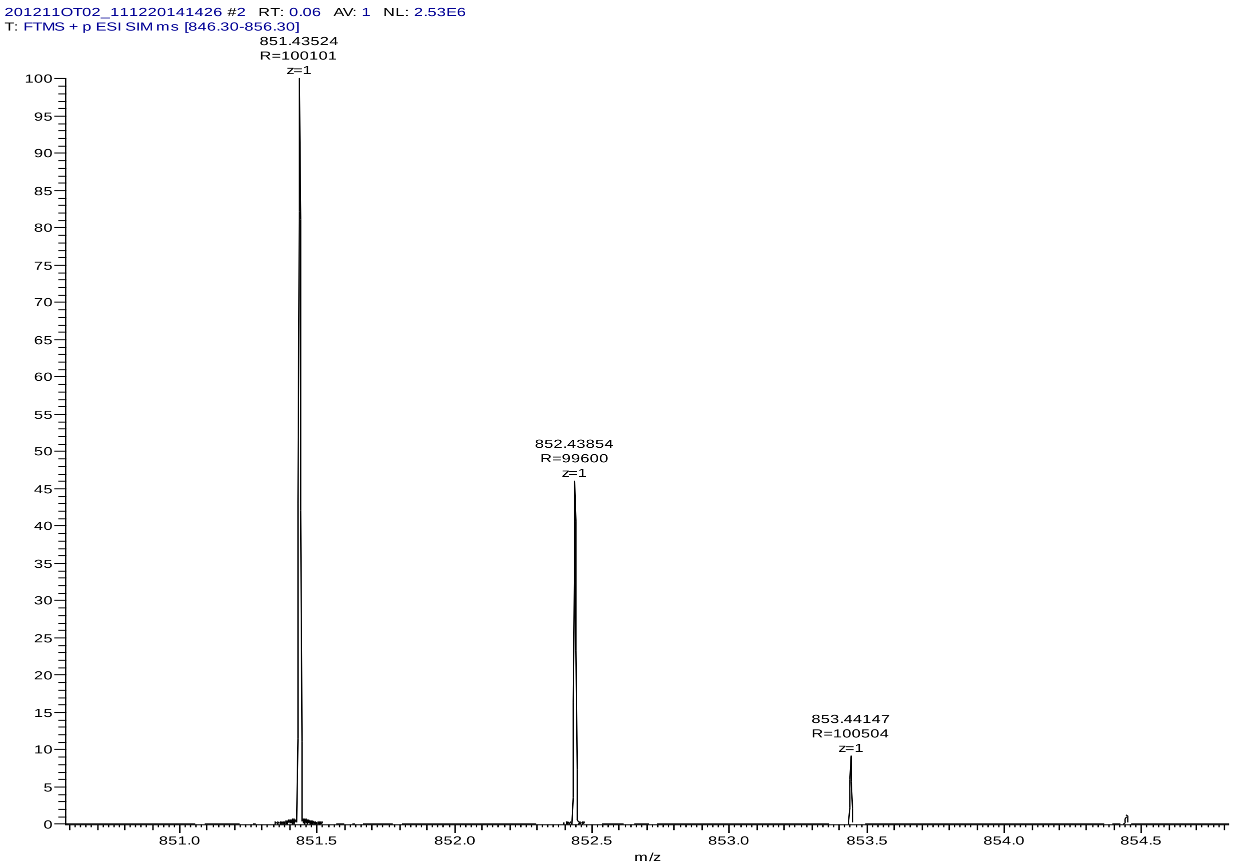

Supplement: Supplementary file 1 [file molecules-18-02598-s001.pdf]
